# The cellular and extracellular proteomic signature of human dopaminergic neurons carrying the LRRK2 G2019S mutation

**DOI:** 10.3389/fnins.2024.1502246

**Published:** 2024-12-12

**Authors:** Felix Knab, Giambattista Guaitoli, Mohamed Ali Jarboui, Felix von Zweydorf, Fatma Busra Isik, Franziska Klose, Anto Praveen Rajkumar, Thomas Gasser, Christian Johannes Gloeckner

**Affiliations:** ^1^Hertie Institute for Clinical Brain Research, Department of Neurodegeneration, University of Tübingen, Tübingen, Germany; ^2^German Center for Neurodegenerative Diseases (DZNE), Tübingen, Germany; ^3^Core Facility for Medical Proteomics, Institute for Ophthalmic Research, Center for Ophthalmology, University of Tübingen, Tübingen, Germany; ^4^Institute of Mental Health, Mental Health and Clinical Neurosciences Academic Unit, University of Nottingham, Nottingham, United Kingdom

**Keywords:** extracellular vesicle, biomarker, induced pluripotent stem cell, LRRK2, Parkinson’s disease

## Abstract

**Background:**

Extracellular vesicles are easily accessible in various biofluids and allow the assessment of disease-related changes in the proteome. This has made them a promising target for biomarker studies, especially in the field of neurodegeneration where access to diseased tissue is very limited. Genetic variants in the LRRK2 gene have been linked to both familial and sporadic forms of Parkinson’s disease. With LRRK2 inhibitors entering clinical trials, there is an unmet need for biomarkers that reflect LRRK2-specific pathology and target engagement.

**Methods:**

In this study, we used induced pluripotent stem cells derived from a patient with Parkinson’s disease carrying the LRRK2 G2019S mutation and an isogenic gene-corrected control to generate human dopaminergic neurons. We isolated extracellular vesicles and neuronal cell lysates and characterized their proteomic signature using data-independent acquisition proteomics. Then, we performed differential expression analysis to identify dysregulated proteins in the mutated line. We used Metascape and gene ontology enrichment analysis on the dysregulated proteomes to identify changes in associated functional networks.

**Results:**

We identified 595 significantly differentially regulated proteins in extracellular vesicles and 3,205 in cell lysates. We visualized functionally relevant protein–protein interaction networks and identified key regulators within the dysregulated proteomes. Using gene ontology, we found a close association with biological processes relevant to neurodegeneration and Parkinson’s disease. Finally, we focused on proteins that were dysregulated in both the extracellular and cellular proteomes. We provide a list of ten biomarker candidates that are functionally relevant to neurodegeneration and linked to LRRK2-associated pathology, for example, the sonic hedgehog signaling molecule, a protein that has tightly been linked to LRRK2-related disruption of cilia function.

**Conclusion:**

In conclusion, we characterized the cellular and extracellular proteome of dopaminergic neurons carrying the LRRK2 G2019S mutation and proposed an experimentally based list of biomarker candidates for future studies.

## 1 Introduction

Parkinson’s disease (PD) is a neurodegenerative disorder affecting millions of patients worldwide and causing both significant morbidity and impaired quality of life (Ou et al., 2021). A combination of genetic and environmental factors has been implicated in triggering PD pathogenesis (Cannon and Greenamyre, 2013). Among the genetic factors associated with PD, variants in the leucine-rich repeat kinase 2 (LRRK2) gene have garnered significant attention. The *LRRK2* G2019S mutation represents the most common genetic cause of familial PD (fPD) and can also be found in sporadic PD (sPD) patients (~2%) due to reduced penetrance of the mutation (Nichols et al., 2005; Zimprich et al., 2004; Schiesling et al., 2008). Genome-wide association studies (GWAS) have further revealed that frequent polymorphisms in the vicinity of LRRK2 are linked to an increased risk of developing sPD (Simón-Sánchez et al., 2009). Various potential roles for LRRK2 on the cellular level of PD pathogenesis have been reported, and they are often associated with the increased kinase activity known to be caused by several of the described LRRK2 mutations (Jaleel et al., 2007).

Although PD is currently diagnosed clinically, the importance of protein-based biomarkers is increasing (Tolosa et al., 2021). Analyses of protein markers such as neurofilament light chain protein or *α*-synuclein in the cerebrospinal fluid (CSF) or blood of patients are becoming more relevant, and recent breakthroughs around α-synuclein seeding assays have the potential to reshape the clinical routine within the next years (Lotankar et al., 2017; Siderowf et al., 2023). However, none of the biomarker assays approaching the clinical routine can pin down the intra-individual PD pathogenesis. With multiple potential LRRK2 inhibitors being developed and entering clinical trials, a biomarker that reflects LRRK2-specific molecular pathology, target engagement, and cellular response is of great interest to the scientific community (Azeggagh and Berwick, 2022). With LRRK2 likely playing a role in some but not all sPD patients, identification of patient subgroups benefitting from inhibitor treatment would further represent an important starting point for the design of future clinical trials. Since its identification as a LRRK2 substrate, Rab proteins have been discussed and their phosphorylation has been utilized as proxies for LRRK2 kinase activity, but it can be assumed that mutated LRRK2 leads to more than just increased phosphorylation of direct target molecules (Azeggagh and Berwick, 2022; Steger et al., 2016). It is therefore important to broaden the portfolio of protein markers indicating LRRK2-related pathophysiology.

In the present study, we aimed to provide an experimentally based list of LRRK2-related protein biomarker candidates. Such candidates should ideally be: (1) functionally relevant in the context of neurodegeneration, (2) functionally linked to LRRK2, (3) reflecting cellular pathogenesis, while also being (4) easily accessible. For this purpose, we used data-independent acquisition mass spectrometry (DIA-MS) to characterize the proteome of extracellular vesicles (EVs) isolated from induced pluripotent stem cells (iPSCs) derived human dopaminergic neurons (hDaNs) and direct cell extracts. This line had previously been generated from an iPSC line derived from a PD patient carrying the LRRK2 G2019S mutation together with an isogenic gene corrected control (Reinhardt et al., 2013a; Reinhardt et al., 2013b).

EVs are membranous vesicles secreted by a variety of cell types into the extracellular space and next to nucleic acids contain intracellular proteins (Colombo et al., 2014). They have proven to be easily accessible from biofluids such as plasma or CSF and throughout the last decade have been intensively studied as sources for biomarkers (Théry et al., 2006; Ciferri et al., 2021). We compared the EV proteome to the cellular proteome of the hDaNs to identify proteins that are dysregulated on both cellular and extracellular levels and to increase the robustness of the findings. Finally, we performed gene ontology analyses and identified LRRK2 interactors among the dysregulated proteins to pin down functionally relevant candidates.

## 2 Materials and methods

### 2.1 Cultivation of human dopaminergic neurons

**Figure 1:**
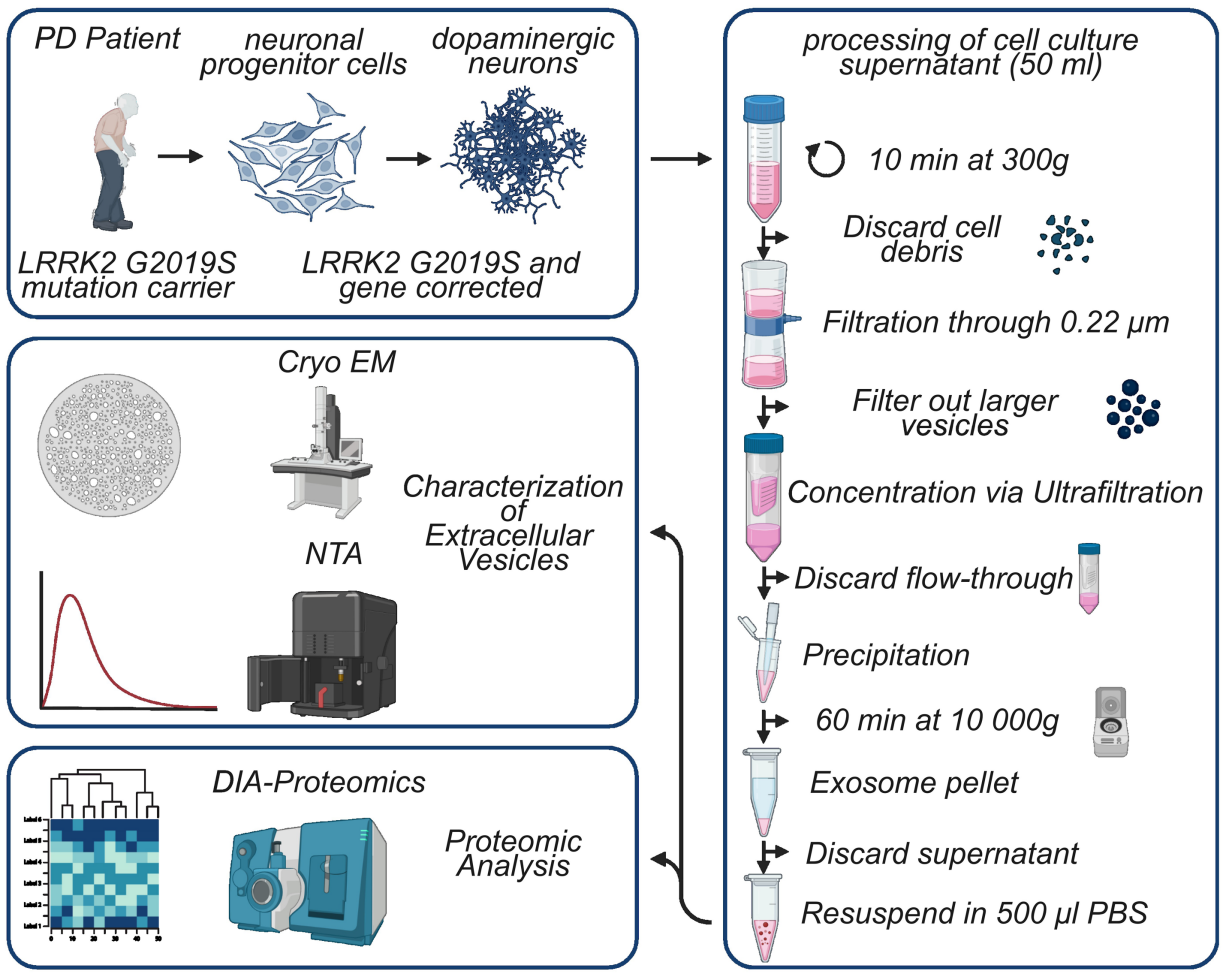
Visual representation of study design and the EV isolation protocol. Neural progenitor cells were generated previously using iPSCs derived from a female PD patient carrying the LRRK2 G2019S mutation. After induction of differentiation over a period of 14 days, cell culture supernatant was collected every 3 days from human dopaminergic neurons, resulting in a total of 36 mL of supernatant. The supernatant was thoroughly processed, and resulting EV samples were characterized using NTA and Cryo-TEM. Finally, proteomes of EV samples and cell lysates of hDaNs were analyzed. Figure generated with biorender.com.

We previously described the generation of patient-derived iPSCs and neuronal progenitor cells (NPCs) from two PD patients carrying the LRRK2 G2019S mutation (Reinhardt et al., 2013a; Reinhardt et al., 2013b). Due to unstable LRRK2 expression of the second line, for the present study only the first line, derived from a female with an age of onset of 40, was used together with the respective gene-corrected control line and will be referred to as L1 GC (gene corrected) and L1 G2019S, formerly referred to as L1. The induction of dopaminergic differentiation of NPCs together with the thorough characterization of the resulting human dopaminergic neurons (hDaNs) has recently been published (Cavarischia-Rega et al., 2024). Briefly, for each genotype, one cryotube of NPCs was thawed and cells were split into three six-well plates. The three six-well plates were expanded independently and functioned as technical replicates for the isolation of EVs and cell lysates. NPCs were cultured and expanded in NPC media (Supplementary Table S1) until they reached 80% confluency, after which differentiation was induced using a media referred to as D7 media (Supplementary Table S1). After 7 days, D7 media were exchanged for maturation media (Supplementary Table S1). On day 14 of the differentiation, 2 mL of maturation media was added to each well of an entire six-well plate. Conditioned cell culture media (CCM) was collected and pooled from one six-well plate after 3 days, resulting in a total of 12 mL of CCM per technical replicate. This was repeated every 3 days until day 23 of the differentiation. CCM from each collection time point was pooled, resulting in a total of 36 mL per technical replicate and was processed further for isolation of EVs (Figure 1).

### 2.2 Isolation of extracellular vesicles

To eliminate cellular debris, CCM collected on days 14 to 23 was centrifuged at 300 g for 10 min after which it was filtered through a 0.22-μm Steriflip filter (Merck, #SCGP00525). To concentrate the CCM to 1 mL, we used the Amicon® Ultracel Centrifugal filters (Merck, #UFC901024), which were centrifuged at 2000 g at 4°C for 30 min. After 1 mL of concentrated CCM was recovered, we transferred it to a 1.5-ml tube and added 500 μL of Total Exosome Isolation Reagent (Thermo Fisher Scientific, #4478359). After overnight incubation at 4° C, samples were centrifuged at 10000 g for 1 h. The supernatant was carefully removed before the tubes were centrifuged for 5 min at 10,000 g. Finally, pellets were washed three times with 1 mL PBS to remove residual traces of the isolation reagent before they were resuspended in 200ul of PBS containing cOmplete protease inhibitor (Sigma-Aldrich, #11873580001) and phosphatase inhibitor (Sigma-Aldrich, #4906837001) (Figure 1).

### 2.3 Characterization of extracellular vesicles

We characterized the extracellular vesicles following the minimal information for studies of extracellular vesicles guidelines (Théry et al., 2018): (1) particle size and concentration were analyzed using nanoparticle tracking analysis (NTA); (2) morphology of vesicles was assessed via cryo-transmission electron microscopy (cryo-TEM); (3) presence of EV protein markers within the proteome of all EV samples was confirmed; and (4) gene ontology (GO) enrichment analysis for the cellular component was performed for proteins detected in all EV samples.

#### 2.3.1 Particle size concentration

Measurements were conducted using the NanoSight NS300 instrument, and NanoSight NTA 3.00068 software was provided by Malvern Panalytical in Kassel, Germany. To optimize the accuracy of the analysis, the samples were diluted in phosphate-buffered saline (PBS) at ratios ranging from 1:100 to 1:500 prior to measurement. Per measurement, five videos with a length of 60 s were recorded.

#### 2.3.2 Cryo-TEM

Cryo-transmission electron microscopy was performed at the Nanoscale and Microscale Research Centre (nmRC) located at the University of Nottingham, UK. The nmRC has previously published a protocol for preparing EV samples for cryo-TEM, which was adapted for this study (Nizamudeen et al., 2021; Nizamudeen et al., 2018); 5 μL of EV sample was added onto each holey carbon TEM grid for 2 min (EM resolutions, Sheffield, UK, #HC300Cu). After the removal of excess solution using filters, EV samples were blotted for a second and then frozen in liquid ethane using a Gatan CP3 plunge freezing unit (Ametek, Leicester, UK). Samples were loaded to an FEI Tecnai G2 12 Bio-twin TEM. Images were obtained using an inbuilt Gatan SIS Megaview-IV digital camera at an accelerating voltage of 100 kV.

#### 2.3.3 Confirmation of the presence of EV marker proteins

We compared the relative abundancy of the EV marker proteins TSG101, CD81, and flotillin-1 and the negative marker calnexin in the proteome of all EV samples to their abundance in hDaNs cell lysates. Relative abundance was calculated by normalizing the protein intensity of a given marker protein to the sum of all protein intensities within the given sample. A two-way ANOVA was conducted to examine the effect of protein of interest and cellular compartment (cellular vs. extracellular) on the relative expression. The same test was used to compare abundancy of marker proteins in EVs derived from either the gene corrected or the LRRK2 G2019 cell line.

In addition, we performed GO enrichment analysis of the most abundant proteins found in the EV samples. We therefore selected the top 100 most abundant unique protein identities in each genotype, identified the overlap between these two, and analyzed the cellular components these proteomes were annotated to. GO enrichment analysis was performed using the *enrichGO* function from the ClusterProfiler package in R and is described in further detail in the statistics section (Yu et al., 2012; R Core Team, 2022).

### 2.4 DIA-based proteomic analysis

#### 2.4.1 Protein extraction

EV proteomes were quantitatively assessed by DIA-based mass spectrometry. For this purpose, the EV fractions were concentrated by lyophilization and re-dissolved in 1x Laemmli buffer. Cell lysates were collected on day 23 by adding 150 μL of a simple lysing buffer (PBS containing 1% of Triton 100x, cOmplete protease, and phosphatase inhibitor) to each well of a six-well plate per replicate and were further processed.

#### 2.4.2 SDS-PAGE and in-gel digestion

Fifty micrograms of total protein was subjected to SDS-PAGE (NuPAGE 10% Bis-Tris gels). The electrophoresis was topped after the sample front reached 1 cm. For visualization of the protein, gels were stained by Coomassie. The lanes were excised, distained, and subjected to in-gel proteolysis by a Trypsin/LysC mix (Promega) following standard protocols (Gloeckner et al., 2009). Extracted and vacuum-dried peptides were subjected to an additional C18-StageTip (Thermo Fisher) pre-cleaning step (Rappsilber et al., 2007). Finally, vacuum-dried samples were dissolved in 0.5% TFA and mixed with 2 μL iRT standard peptide mix (Biognosis).

#### 2.4.3 LC–MS/MS analysis

Mass spectrometry analysis was performed on an Ultimate3000 RSLC system coupled to an Orbitrap Tribrid Fusion mass spectrometer (Thermo Fisher Scientific). Tryptic peptides were loaded onto a μPACTM Trapping Column (#COL-TRPNANO16G1B2, Fisher Scientific) at a flow rate of 10 μL per min in 0.1% tri-fluoroacetic acid in HPLC-grade water. Peptides were eluted and separated on a 50-cm μPACTM C18 nano-LC column (#COL-NANO050G1B, Fisher Scientific) by a linear gradient from 2 to 30% of buffer B (80% acetonitrile and 0.08% formic acid in HPLC-grade water) in buffer A (2% acetonitrile and 0.1% formic acid in HPLC-grade water) at a flow rate of 300 nL per min. The remaining peptides were eluted by a short gradient from 30 to 95% buffer B; the total gradient run was 120 min. Spectra were acquired in DIA (data-independent acquisition) mode using 50 variable-width windows over the mass range 350–1,500 m/z, MS2 scan range was set from 200 to 2000 m/z.

#### 2.4.4 Primary data analysis

Data analysis was performed using DIA-NN (ver. 1.8.1) activating the following options: Trypsin/P as an enzyme, “FASTA digest for liberty-free search/ library generation” (database: a human subset of SwissProt 2021_04, 20,375 entries), and “Deep learning-based spectra, RTs an IMs predication.” (Demichev et al., 2020) In addition, the *match between runs* (MBR) option was activated. Carbamidomethylation was set as a fixed modification, and N-terminal methionine excision was allowed. Cross-run normalization was done RT-dependent, and smart profiling was used for library generation. In addition, a heuristic model for protein inference was used. Mass accuracies and window widths were determined/detected by the algorithm. In addition, isotopologues were considered and no shared spectra were allowed. A high-precision robust LC separation was assumed for quantification. The precursor FDR was set to 1%.

### 2.5 Experimental design and statistical rationale

From each genotype, we isolated 36 mL of CCM per differentiation (3x LRRK2 G2019S, 3x LRRK2 Gene Corrected). Each of the 36 mL of CCM resulted from an individual six-well plate of iPSC-derived human dopaminergic neurons that had been cultured as described above. All resulting EV samples were analyzed via NTA and DIA. Cryo-TEM images were made using 100 μL of one LRRK2 G2019S and one LRRK2 GC EV sample derived from an additional differentiation that was not further included in the study.

Downstream analysis on maxLFQ values (DIA-NN unique genes matrix) was performed with Perseus (ver. 1.6.7.0) (Tyanova et al., 2016). Data were log2 transformed to facilitate the identification of proportional changes in protein abundance between different groups. This transformation helps normalize the data and stabilize the variance, making it more suitable for subsequent statistical analysis. After categorical annotation, only IDs with three valid values in at least one biological group were accepted. Missing data were imputed separately for each biological group using the normal distribution; the width and downshift were set to 0.8 and 1.3, respectively. Significant changes were detected by a two-sided Student’s *t*-test using a permutation-based FDR (S0 = 1, FDR = 0.05 with 250 randomizations).

Statistical analysis of the NTA data, namely unpaired *t*-test on differences of particle yields and sizes between the genotypes, was performed using GraphPad Prism software, version 9.3.0. (La Jolla, CA, United States). QQ plots were used to assess the normality of the data. The mean relative abundance of marker proteins TSG101, flotillin-1, CD81, and calnexin in hDaNs cell lysates was calculated using protein intensities within the proteomics dataset. Then, for each EV sample a log2 fold change (log2 fc) value of that mean abundance was calculated.

#### 2.5.1 Data visualization

For further data analysis and visualization, various functions from R-Studio, version 4.3.0 were used (R Core Team, 2022); principal component analysis (PCA) of the six EV samples and six cell lysates was performed using the built-in *prcomp* function and using the data of proteins identified in all 12 samples. For the generation of heatmaps, proteins that were not present in all samples were excluded. The built-in *scale* function was used to normalize the data. When plotting data from hDaNs and EVs in one heatmap, intensities were normalized separately for each sample type; for downstream analysis, a fold-change cutoff of > ± 1.5 was applied. Proteins that did not cross this threshold were not further considered, even if they passed statistical testing of differential expression.

#### 2.5.2 Functional enrichment and interaction networks

The Metascape platform was used for an initial scan of dysregulated proteomes (Zhou et al., 2019). Briefly, Metascape integrates multiple enrichment and functional analysis approaches such as the string database, KEGG pathways and GO processes. It identifies all statistically enriched terms from either of the included enrichment analyses. Then, significant terms are clustered based on similarity. For each cluster, the term with the best *p*-value is selected as its representative label. A subset of 20 clusters is automatically selected and converted into a network layout where terms are represented by a node and edges connect terms passing a similarity threshold. Finally, Metascape uses the Molecular Complex Detection (MCODE) algorithm to identify densely connected clusters within the dysregulated protein networks of either the EV or the cellular proteome (Hogue and Groll, 2001). Briefly, MCODE detects highly interconnected regions within protein–protein interaction networks, which may correlate to functional modules. For the sake of clarity, we filtered out networks with less than four nodes. For the visualization of protein–protein interaction networks, we used the *string_db* function from the *string_db* package (Szklarczyk et al., 2023).

#### 2.5.3 Targeted GO enrichment analyses

GO enrichment analysis was performed using the *enrichGO* function from the *ClusterProfiler* package (Yu et al., 2012); if not stated otherwise, proteins that were either up- or downregulated were analyzed separately. The *p*-value cutoff was set to 0.05, FDR correction was performed using the Benjamini–Hochberg procedure to correct for multiple testing and adjusted *p*-values will be reported (Benjamini, 1995). To filter out GO terms relevant to the research question, we filtered for CNS-related GO terms using the *grep* function in R and a customized list of terms (Supplementary Table S2). If applicable, the top 15 GO terms sorted by *p*-value were visualized using a semantic scatter plot with a maximum similarity matrix being calculated to indicate the semantic proximity of identified GO terms. If applicable, we further visualized the top three GO terms and their annotated proteins in a network plot.

The analytical platform Omics Playground^1^ was used for proteomics data exploration and integration. We used the find biomarkers feature to identify the most important proteins that could be used as predictors for biological group GC or G2019S. The analysis is based on calculating importance scores using multiple machine learning algorithms such as random forest, XGboost, sPLS, and correlation analysis.

Finally, we used Cytoscape software, version 3.8.2, and String database to identify the LRRK2 interactome. The physical subnetwork was plotted, and a confidence score cutoff was set at the default of 0.4 (Shannon et al., 1971; Szklarczyk et al., 2015).

### 2.6 Semi-automated literature review

To identify promising biomarker candidates, we performed semi-automated literature research on the final set of proteins identified to be dysregulated in both EVs and cells. The search was conducted via the *entrez_search* function from the *rentrez* package in R and applied to the PubMed database to retrieve PubMed IDs associated with the identified proteins and the research question. This function returned PubMed IDs based on the defined protein list and term queries. We selected the terms “Parkinson’s disease” and “LRRK2.”

## 3 Results

### 3.1 Characterization of hDaNs derived extracellular vesicles

**Figure 2:**
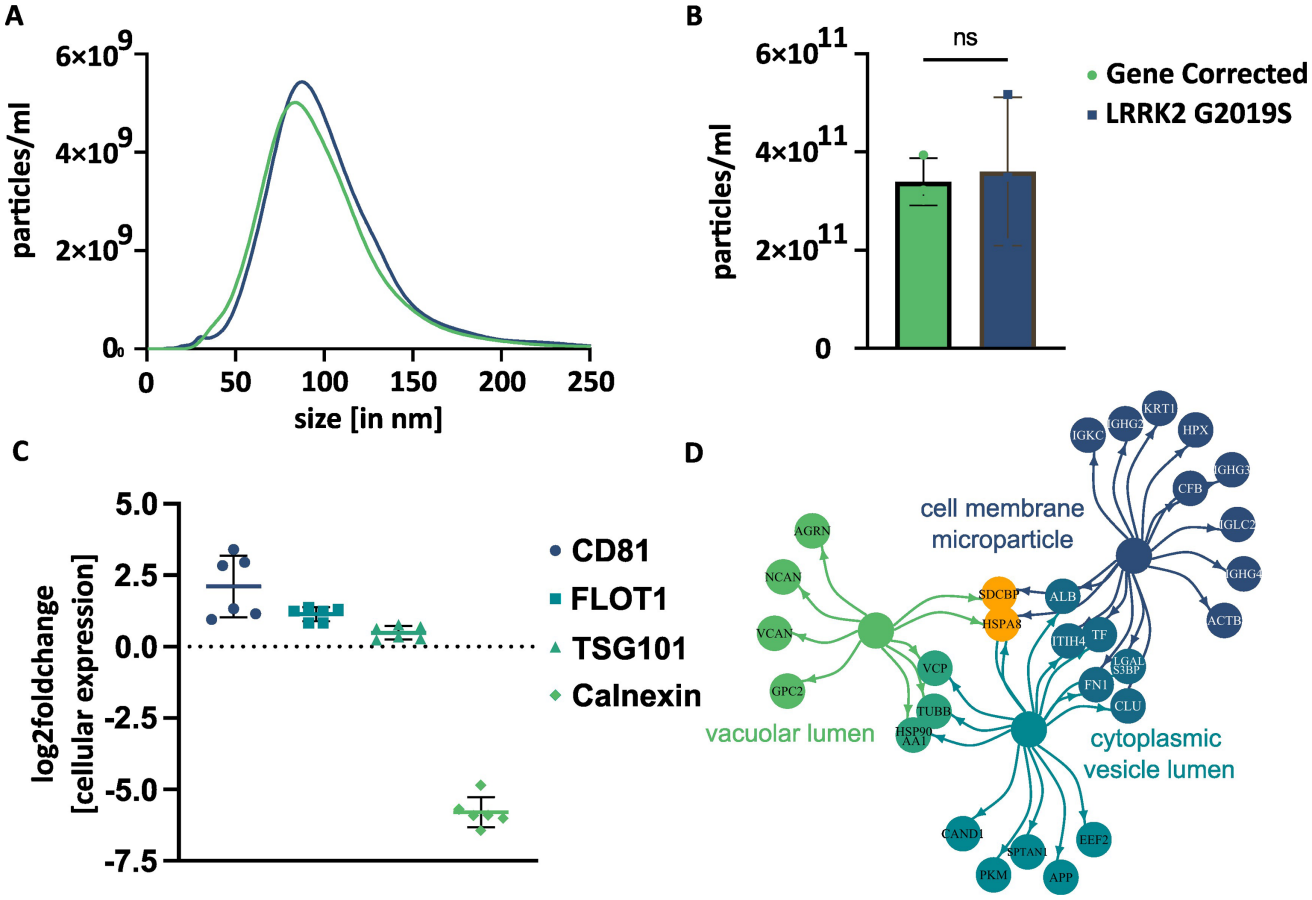
Basic characterization of EVs isolated from hDaNs derived cell culture supernatant. NTA was performed on 3 EV samples per genotype. The size of EVs ranged from 30 to 220 nm with a peak approximately 80 to 90 nm **(A)**. No significant difference in particle yield between genotypes was detected using an unpaired *t*-test. Error bars indicate standard deviation **(B)**. Relative abundance of EV marker proteins was calculated using protein intensities within the different proteomics datasets. EV markers CD81, flotillin-1 (FLOT1), and TSG101 were more abundant in EVs, while negative marker calnexin was clearly more abundant in cells **(C)**. GO enrichment analysis was performed for cellular components on the most abundant proteins detectable in all six EV samples. Depicted terms were among the top ten significantly associated cellular components. The color of the circles indicates to which term the given proteins were annotated to. If a protein was annotated to two terms, the depicted color is a mix of both. Orange proteins were annotated to all three terms **(D)**.

NTA confirmed the presence of particles with a size of 30 to 220 nm, with peaks approximately 80–90 nm (Figure 2A). The mean particle size was 98.5 nm (SD: ±1.1) in EVs from L1 GC and 103.2 nm (SD: ±8.2) in EVs from L1 G2019S. The unpaired *t*-test showed no statistically significant differences between the two lines (difference between means: 4.7, *t* = 0.99, df = 4, *p* = 0.376). The mean particle yield per line and batch of CCM were 3.4×10^11^ EVs (SD: 4.8×10^10^) in L1 GC and 3.6×10^11^ EVs (SD: 1.5×10^11^) in L1 G2019S. Again, the unpaired *t*-test did not show any significant difference between lines (difference between means: 2.1×10^11^, *t* = 0.23, df = 4, *p* = 0.184) (Figure 2B).

We validated the isolation protocol by comparing the relative abundance of EV marker proteins, CD81, flotillin-1, and TSG101 and the relative scarcity of calnexin, in the proteome of EVs and cell lysates (Figure 2C). The relative abundance of CD81 (mean log2 fc compared to cells: 2.1, SD: 1.1, *n* = 6) and flotillin-1 (mean log2 fc: 1.1, SD: 0.2, *n* = 6) was higher in EVs than in hDaNs. The abundance of TSG101was slightly higher in EVs (log2 fc: 0.5, SD: 0.2, *n* = 6) than hDaNs, while levels of calnexin (log2 fc: −5.8, SD: 0.5, *n* = 6) were clearly higher in hDaNs than EVs.

We additionally compared the relative abundance of marker proteins in EVs and cell lysates and found a statistically significant interaction between protein and cellular compartment on the relative expression [*F*(3, 49) = 162.6, *p* < 0.0001]. After correction for multiple comparisons using the Šidák test, we found significant differences in the abundance of CD81 in EVs compared to cells (*t* = 7.7, df = 40, *p* < 0.0001) (Supplementary Figure S1A). In contrast, the abundance of calnexin was significantly increased in cells (*t* = 20.5, df = 40, *p* < 0.0001). There was no significant difference in the abundance of flotillin 1 or TSG101 when comparing EVs and cell lysates.

Both genotype and protein of interest did have an effect on the relative expression [*F*(3, 16) = 33.3, *p* < 0.0001]. After correction for multiple comparisons using Šidák test, we found that EVs derived from L1 G2019S seemed to express CD81 more than EVs derived from L1 GC (*t* = 11.4, df = 16, *p* < 0.0001), whereas all other EV markers were not significantly different (Supplementary Figure S1B).

To further assess the proteome of the EV samples and confirm their vesicular nature, we performed GO enrichment analysis with the most abundant proteins. Of the 100 proteins selected per genotype, 77 proteins were among the most abundant in EVs from both L1 GC and L1 G2019S. Out of the 51 significantly associated GO terms (Supplementary Table S3), many were associated with either vesicles or the endosomal–lysosomal pathway. Among the top ten terms were “*cell membrane microparticle*” (associated proteins: 17/77, *p* < 0.0001), “*cytoplasmic vesicle lumen*” (16/77, *p* < 0.0001), and “*vacuolar lumen*” (9/77, *p* < 0.0001) (Figure 2D).

In Cryo-TEM, particles appeared as solitary, spherical, and membrane-encapsulated structures, in line with previous reports of EVs (Raposo and Stoorvogel, 2013). We did not find any morphological differences between the two genotypes (Supplementary Figure S2).

### 3.2 Proteomic analysis

#### 3.2.1 Quantitative analysis of proteomes

**Figure 3:**
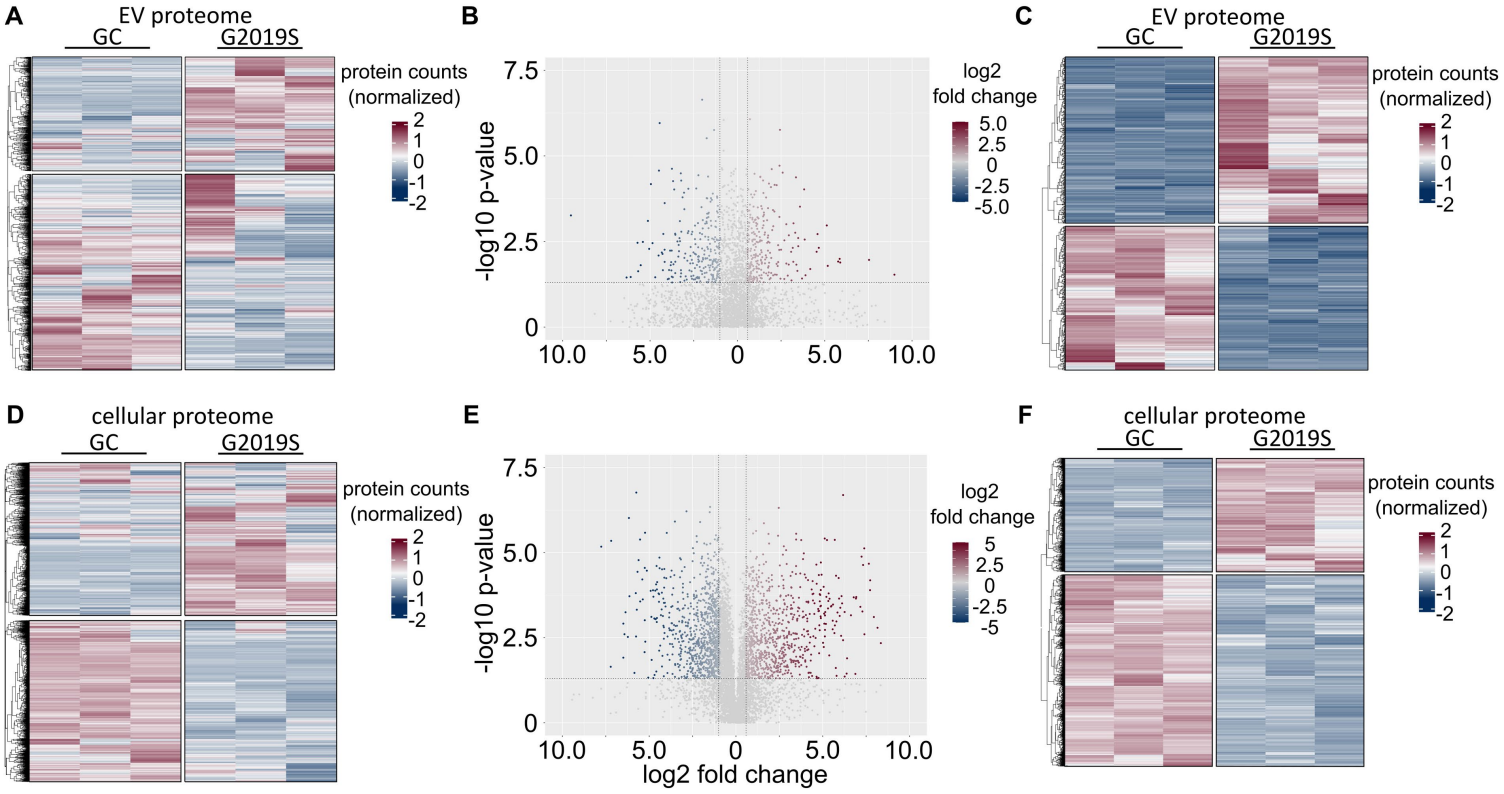
Proteomic signature of EVs and cell lysates derived from hDaNs carrying the LRRK2 G2019S mutation. In EVs isolated on days 14 to 23 of the differentiation, a total of 2,634 proteins could be identified in all six samples. When normalizing protein counts, separation along genotype becomes apparent **(A)**. Volcano plot visualizes log2 fold change levels of proteins found in L1 G2019S compared to L1 GC. The dotted horizontal line visualizes the *p*-value cut-off at 0.05, whereas vertical lines indicate the > ± 1.5 fold-change threshold. Of the 595 significantly dysregulated proteins, 484 passed this cutoff **(B)**. Heatmap of differentially expressed proteins in EV samples **(C)**. In cell lysates of hDaNs isolated on day 23 of the differentiation, 5,833 proteins were found **(D)**. A total of 3,205 proteins were significantly dysregulated, whereas 1833 passed the fold-change threshold **(E)**. Heatmap of differentially expressed proteins in cell lysates **(F)**.

Quantitative proteomics was performed using the data-independent analysis (DIA) approach. Three technical replicates were analyzed for EV as well as for the corresponding cell lysates of the same differentiations. Using DIA-NN, libraries were created by the extraction of pseudo-MSMS spectra directly from the DIA runs (Demichev et al., 2020). By this approach, a total of 2,634 unique proteins were consistently detectable in all six EV samples. The resulting heatmap separated genotypes by protein abundance (Figure 3A). In total, 595 proteins were significantly dysregulated (n_upregulated_ = 318; n_downregulated_ = 277) in L1 G2019S compared to L1 GC (Figures 3B,C). Of those, 484 passed the fold-change threshold (n_upregulated_ = 275; n_downregulated_ = 209). In the cell lysates of hDaNs, a total of 5,833 unique proteins were consistently detectable in all six samples (Figure 3D). In total, 3,205 proteins were significantly dysregulated (n_upregulated_ = 1,190; n_downregulated_ = 2015) in L1 G2019S compared to L1 GC (Figures 3E,F). Of those, 1833 passed the fold-change threshold (n_upregulated_ = 907; n_downregulated_ = 926).

#### 3.2.2 Identification of protein–protein interaction networks and functional enrichments

**Figure 4:**
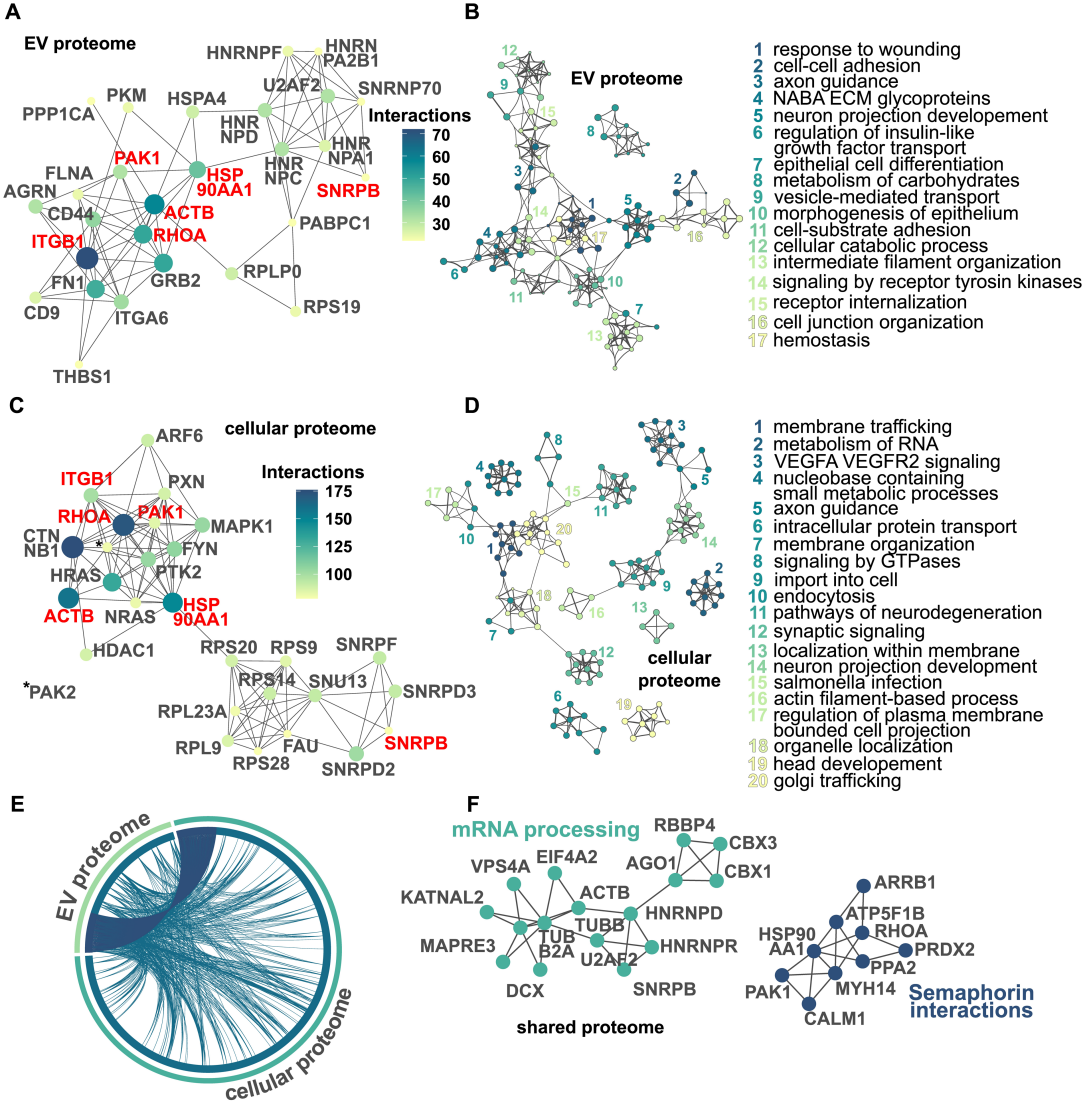
Visualization of dysregulated protein networks and pathways in the EV and cellular proteomes. Protein–protein interaction network within the dysregulated EV proteome displaying the top 25 proteins based on a number of interactions. Nodes represent proteins, with their color corresponding to the number of interactions, and edges representing protein–protein interactions. The size of the nodes also indicates the number of interactions with larger nodes indicating more interactions. Protein names in red highlight shared interactors in the EV and cellular proteome. As multiple proteins are tied at position 25, the actual number of nodes depicted is 27 **(A)**. Network of enriched terms within the EV proteome using the Metascape analysis pipeline. Here, a subset of the enriched terms is depicted with each node representing one term. Similar terms are clustered together and have the same color. The name of each cluster is based on the term with the smallest *p*-value within its respective cluster. For clarity, only networks with at least two edges were selected **(B)**. Protein–protein interaction network within the dysregulated cellular proteome. Again, multiple proteins are tied at position 25 and the actual number of nodes depicted is 27. Proteins written in red represent interactors found in both the EV and cellular proteome to be among the top 25 **(C)**. Network of enriched terms within the cellular proteome **(D)**. The chord diagram displays the overlaps between the EV and the cellular proteomes. Edges in dark blue depict overlaps on the protein level, while edges in dark teal indicate proteins annotated to the same enriched ontology term **(E)**. MCODE-based protein–protein interaction network visualizing networks identified within the shared dysregulated proteome. For each cluster, the term with the best *p*-value is selected as its representative label. For the sake of clarity, only networks with more than three nodes were depicted **(F)**.

Using the string database, we visualized the top 25 proteins that had the most interaction partners within either the dysregulated EV or cellular proteome, respectively (Figures 4A,C). Out of these 25, a total of six proteins were identified in both proteomes: ACTB, HSP90AA1, ITGB1, PAK1, RHOA, and SNRPB. Then, using Metascape, we visualized and identified multiple networks of functionally enriched terms (Figures 4B,D). Within the dysregulated EV proteome, we found, among others, the terms “response to wounding,” “axon guidance,” and “vesicle-mediated transport.” In the dysregulated cellular proteome, we identified terms such as “membrane trafficking,” “intracellular protein transport,” and “pathways of neurodegeneration.” Then, we visualized how proteins from either datasets were overlapping (Figure 4E). First, we identified those proteins that were present in both datasets. Second, we visualized those proteins that were functionally connected to each other. As shown in Figure 4E, although only a fraction of dysregulated proteins is shared between both datasets, the cellular and extracellular proteomes appear to be functionally closely related. Finally, using MCODE on the shared dysregulated proteome, we visualized functional clusters and the corresponding protein–protein interaction networks (Figure 4F). Here, we identified two networks with the corresponding labels “mRNA processing” and “semaphorin interactions.”

#### 3.2.3 Functional enrichment analyses using gene ontology

**Figure 5:**
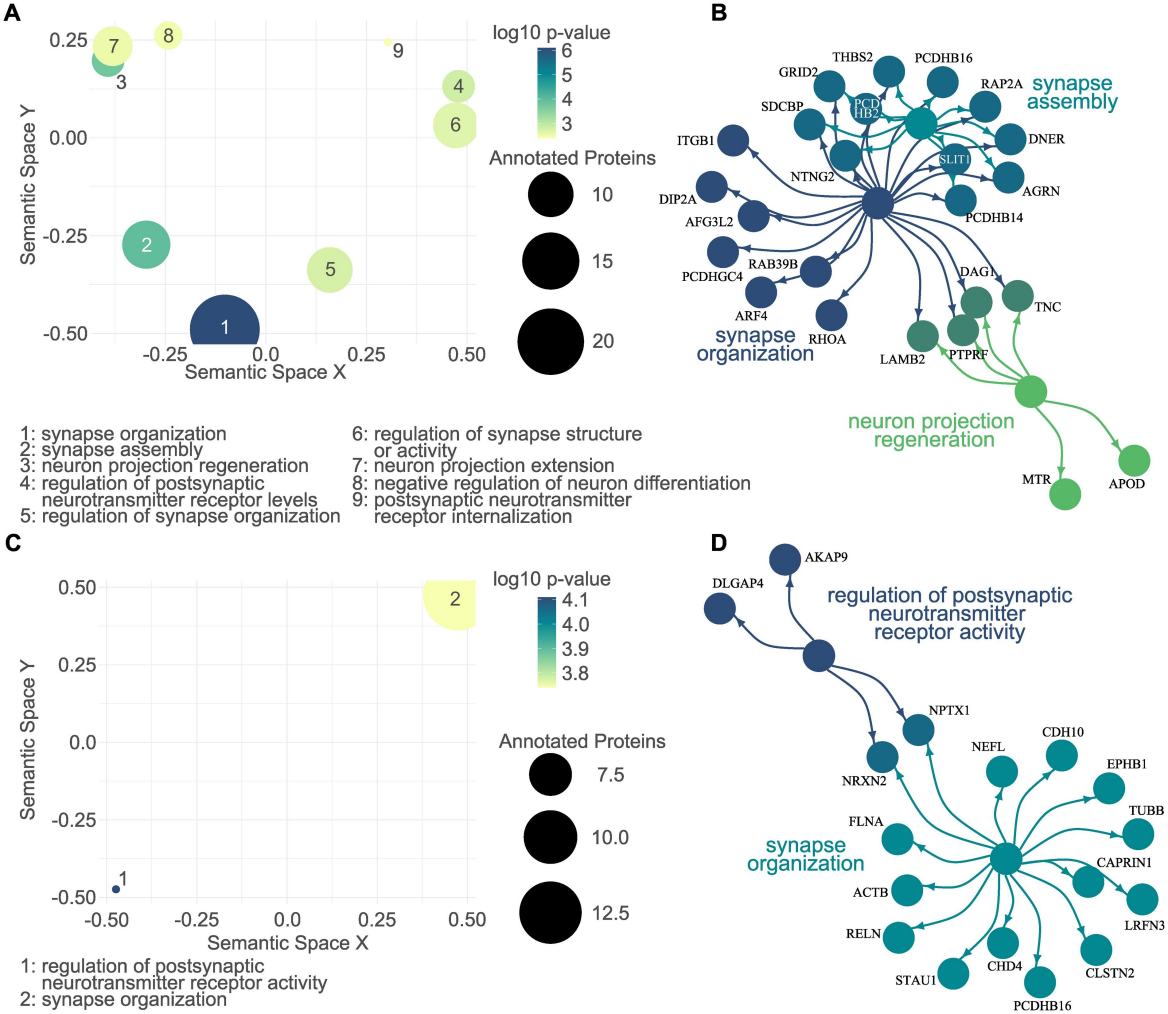
CNS-related gene ontology enrichment analysis of the L1 G2019S EV proteome. A semantic scatter plot visualizes the identified GO terms in a two-dimensional semantic space. Terms that are semantically related are depicted more closely to each other. The size of the dots represents a number of annotated proteins, while the color depicts the log10 *p*-value. A total of nine CNS-related GO terms were significantly associated with the proteins upregulated in L1 G2019S EVs **(A)**. Of those, the top three were visualized together with their annotated proteins. The color of the dots indicates the respective GO term. Proteins that were annotated to more than one term are colored in a mix of the respective colors **(B)**. GO enrichment analysis was repeated for the proteome downregulated in L1 G2019S EVs **(C)**. Here, only two CNS-related GO terms were identified and plotted in **(D)**, a network plot.

Functional enrichment analyses of the dysregulated proteomes were performed separately for up- and downregulated proteins. For the sake of stringency, we focused on the *biological processes* significantly associated with the dysregulated proteomes. Among the significantly associated GO terms for the proteins upregulated in L1 G2019S EVs were *synapse organization*, *synapse assembly*, and *neuron projection regeneration* (Figures 5A,B; Supplementary Table S4). A total of two CNS-related GO terms were associated with the downregulated EV proteome, namely *regulation of postsynaptic neurotransmitter receptor activity* and *synapse organization* (Figures 5C,D; Supplementary Table S5).

**Figure 6:**
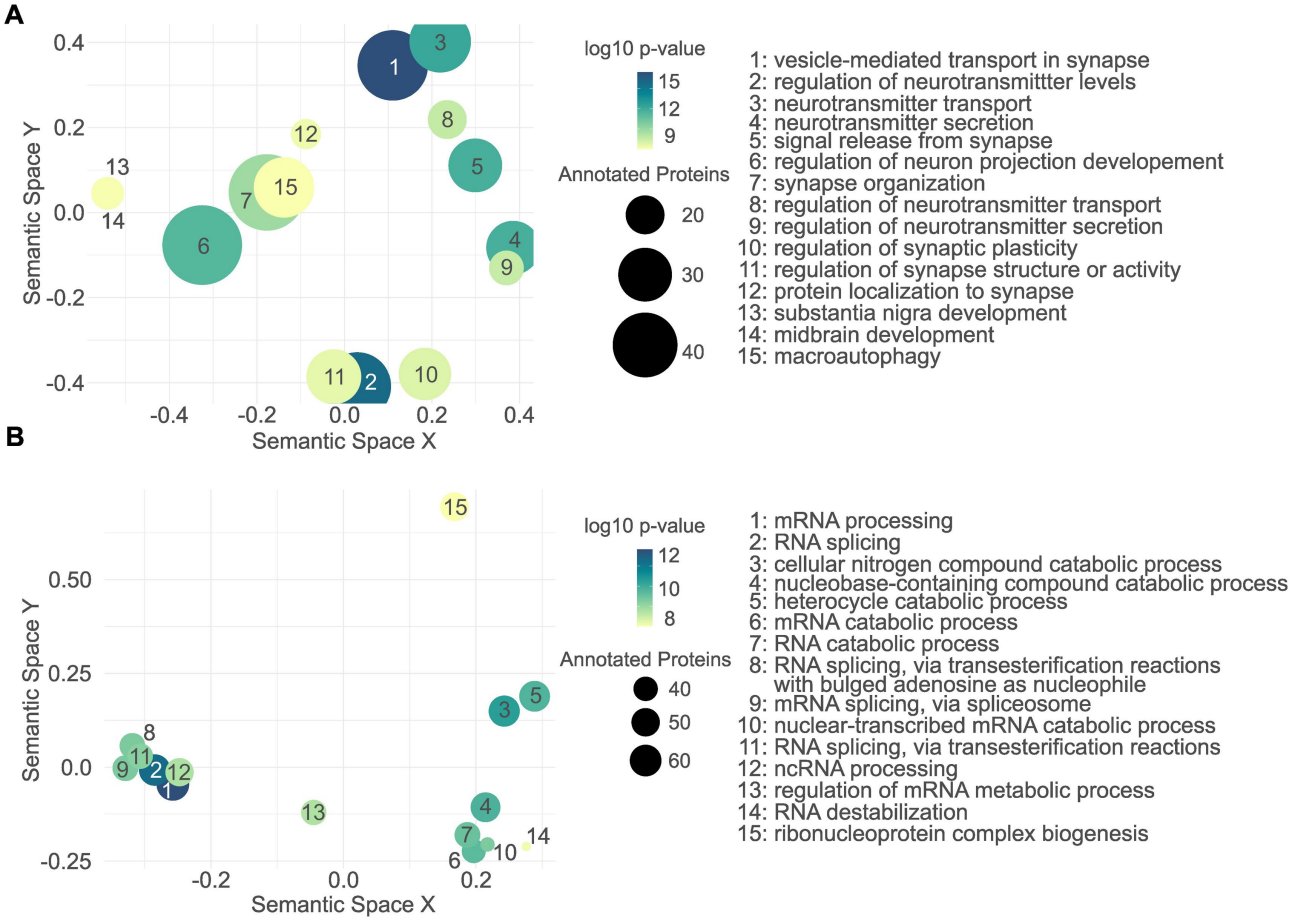
Gene ontology enrichment analysis of the L1 G2019S cellular proteome. Semantic scatter plot of CNS-related GO terms found to be significantly associated with the upregulated proteins in L1 G2019S hDaNs **(A)**. In contrast to the upregulated proteins, no CNS-related GO term was significantly annotated to the downregulated proteome **(B)**. Instead, the unfiltered GO terms are plotted, most of which seem to indicate changes in RNA-related processes.

As for the cellular proteome, among others, upregulated proteins were significantly associated with *vesicle-mediated transport in synapse*, *regulation of neurotransmitter levels*, and *neurotransmitter transport* (Figure 6A; Supplementary Table S6). In contrast, the downregulated proteins were not significantly associated with any CNS-related GO terms. We therefore did not apply the filtering step and plotted the top 15 terms resulting from the total GO enrichment analysis (Figure 6B; Supplementary Table S7), among those were *RNA processing*, *RNA splicing*, and *cellular nitrogen compound catabolic process*.

#### 3.2.4 Analysis of commonly dysregulated proteins

**Figure 7:**
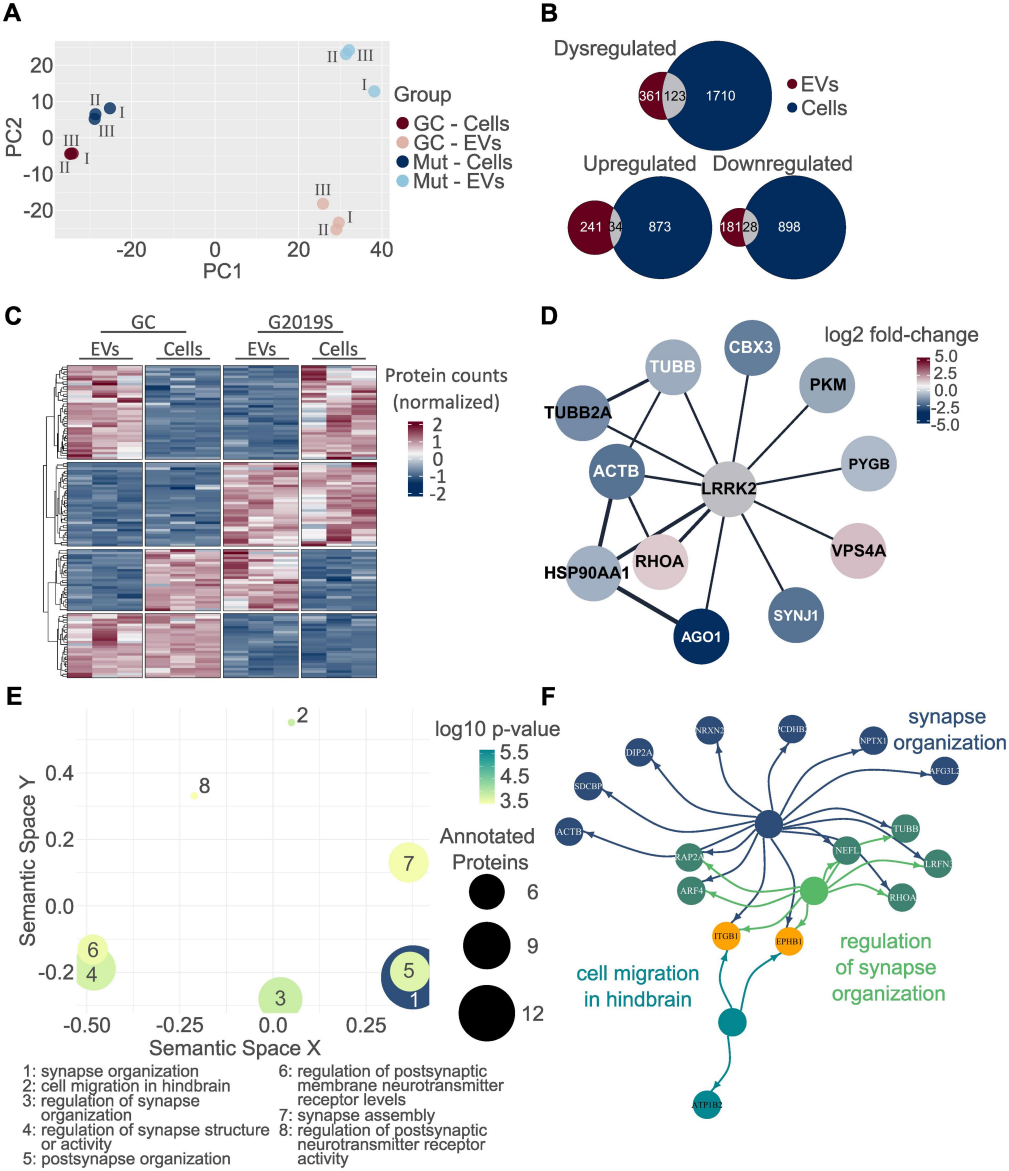
Analysis of commonly dysregulated proteins. PCA was performed on all proteins identified in both EVs and cell lysates. EVs are clearly separated from cell lysates along PC1, while PC2 separates along genotype. In particular, differences between proteomes seem to be larger in EVs than cells **(A)**. A total of 123 proteins were dysregulated on both EVs and cells as indicated by the overlap colored in gray. The direction of dysregulation was the same in EVs and cells for 34 and 28 proteins, respectively **(B)**. The heatmap visualizes the normalized protein intensities in each technical replicate and in EVs and cell lysates. Normalization was performed for each compartment (EVs vs. cell lysates) separated. Interestingly, a considerable fraction of proteins was not dysregulated in the same direction when comparing the cellular and extracellular proteome of the same genotype **(C)**. Using Cytoscape and the String database, out of the 123 proteins dysregulated in both the EV and cellular proteome, a total of 11 proteins appear to be within the LRRK2 interactome. Colors are based on the fold-change expression in G2019S EVs **(D)**. GO analysis was performed on all 123 proteins dysregulated in both EVs and hDaNs **(E)**. The top three hits are visualized together with their annotated proteins. Again, the color of the circles indicates toward which of the GO terms a given protein was annotated. Proteins annotated to two terms are colored in a mixture of the color of the term, while orange indicates annotation toward all three terms **(F)**.

As a final step, we analyzed the proteins that were dysregulated in both the proteome of EVs and hDaNs cell lysates to increase the robustness of the findings. First, we performed PCA using data from all proteins identified in both EVs and cell lysates (Figure 7A). PC1 explained 53.1% of the variability and separated EVs from cell lysates, whereas PC2 explained 13.5% of the variability and separated genotypes. In particular, data from the two cellular proteomes, that is, L1 GC and L1 G2019S, clustered closer together compared to the EV proteomes. Then, we looked at the overlap of dysregulated proteins (Figure 7B). Of the 484 dysregulated proteins in EVs and 1833 dysregulated proteins in hDaNs, a total of 123 proteins were found to be dysregulated in both; 34 proteins were found to be upregulated, whereas 28 were downregulated in both sample types. The remaining 61 proteins were dysregulated in opposite directions, for example, upregulated in cells but downregulated in EVs and vice versa (Figures 7B,C). Using Cytoscape and the String database, we identified and visualized those proteins among the 123 that were described to be within the LRRK2 interactome (Figure 7D). Finally, we performed GO enrichment analysis using all 123 proteins as input and identified, among others, the GO terms *synapse organization*, *cell migration in hindbrain*, and *regulation of synapse organization* to be significantly annotated (Figures 7E,F; Supplementary Table S8).

#### 3.2.5 Semi-automated literature review

Out of the 123 proteins, for 76 proteins we found at least one PubMed ID when using the protein name in combination with “*AND Parkinson’s disease*,” totaling up to 608 publications. For the combination of a protein and the term “*AND LRRK2*,” we found 57 publications for 22 proteins, all of which were also identified using the “*AND Parkinson’s disease*” term (Supplementary Figure 3). Supplementary Table S9 shows all 123 proteins and their respective count of publication. Out of these 22 proteins, we identified 10 interesting candidates based on the available literature (Table 1).

**Table 1:** Overview of functionally relevant biomarker candidates.

Protein name	Gene name	Log2 fc EVs	Log2 fc hDaNs	String LRRK2 Interactome	Function (Stelzer et al., 2016; Safran et al., 2021)	Context
Actin Beta	ACTB	−2.3	6.6	Yes	Crucial and ubiquitously expressed protein of the cytoskeleton	Enriched in synapses, role of LRRK2 in cytoskeleton reorganization (Toh et al., 2021)
Argonaute RISC Component 1	AGO1	−3.6	−1.6	Yes	RNA interference, e.g., repression of mRNA translation, binding to miRNAs	Dysregulated RNA processing in PD, LRRK2 driven miRNA signatures (Braunger et al., 2024; Gehrke et al., 2010)
Annexin A1	ANXA1	2.4	2.1	No	Inhibition of phospholipase A2	Role in neuroinflammation (Darvish et al., 2021)
CD44	CD44	3.8	3.2	No	Cell-surface protein, role in cell–cell interaction and migration	Role in neuroinflammation, upregulated in CSF of LRRK2 PD (Pasterkamp and Giger, 2009; Karayel et al., 2022)
Galactosylceramidase	GALC	−3.9	−1.0	No	lysosomal protein involved in sphingolipid metabolism	Related to GBA, influence on lysosomal membranes (Senkevich et al., 2023)
Peroxiredoxin 2	PRDX2	0.9	−1.0	No	antioxidant enzyme	Phosphorylated by LRRK2, leading to loss of dopaminergic neurons (Chua et al., 2020)
Ras Homolog Family Member A	RHOA	1.0	1.0	Yes	small GTPases, promotes cytoskeleton reorganization	Remodeling of synaptic cytoskeleton, linked to damage of dopaminergic neurons (Tolias et al., 2011; Schmidt S. I. et al., 2022)
Sonic Hedgehog Signaling Molecule	SHH	1.0	2.9	No	Key signaling molecule	Disruption of SHH signaling in LRRK2 as a mechanism of neuronal vulnerability (Schmidt S. et al., 2022; Khan et al., 2021)
Synaptojanin 1	SYNJ1	−2.3	1.3	Yes	Synaptic transmission and membrane trafficking	Phosphorylated by LRRK2, deregulating synaptic vesicle trafficking (Pan et al., 2017)
Tubulin Beta Class I	TUBB	−0.9	0.4	Yes	Structural component of microtubules	LRRK2 leading to microtubule instability (Law et al., 2014)

Based on their functional roles in PD and their association to LRRK2, we selected 10 potential biomarker candidates.

## 4 Discussion

In the present study, we aimed to identify protein-based biomarker candidates that are functionally relevant in the context of PD and are linked to LRRK2. This goal represents an important step toward developing targeted therapies, especially with LRRK2 inhibitors rapidly approaching clinical trials (Azeggagh and Berwick, 2022). We isolated EVs from hDaNs carrying the LRRK2 G2019S mutation and an isogenic, gene-corrected control and used DIA-proteomics to analyze the genotype-specific cellular and extracellular proteomic signatures. Finally, we performed gene ontology analysis for both the cellular and extracellular proteomes and thoroughly reviewed the literature to identify promising candidates for future pre-clinical or clinical biomarker studies.

As a result of the GO enrichment analysis on dysregulated proteins found both in EVs and cell lysates, we identified two major biological processes to be dysregulated in the LRRK2 G2019S cell model, one of which was the structural and functional integrity of synapses. Synapses have extensively been described to potentially play a critical role in the development of neurodegenerative diseases, including LRRK2-linked PD. For example, one study showed LRRK2-dependent regulation of the pre- and postsynaptic morphology via the interaction with microtubule-associated-protein-like targets (Lee et al., 2010). LRRK2 was also shown to interfere with endocytosis of synaptic vesicles, thus affecting neurotransmission, and increased LRRK2 kinase activity was shown to affect striatal dopamine release and uptake in an LRRK2 G2019S mouse model (Arranz et al., 2015; Li et al., 2010). Along these lines of evidence is the identification of dysregulated levels of synaptojanin 1 (SYNJ1), tubulin beta class I (TUBB), cytoskeletal protein actin ß (ACTB), and Ras homolog family member A (RHOA) in the cell model.

SYNJ1, which is involved in synaptic transmission and membrane trafficking, interacts with several proteins playing a key role in synaptic vesicle recycling processes (Bae and Kim, 2017). It was described to be directly phosphorylated by LRRK2, suggesting a close interplay of these two proteins in deregulating the trafficking of synaptic vesicles (Pan et al., 2017). TUBB, which forms a structural component of microtubules, was also shown to interact with LRRK2, resulting in affection of microtubule stability (Law et al., 2014). As neuronal cells are highly dependent on an effective intracellular transport provided by microtubules, their disruption is likely an early event in many neurodegenerative disorders (De Vos et al., 2008). RHOA, on the other hand, likely controls the actin cytoskeleton’s rearrangement and is crucial for coordinating the formation and remodeling of synapses (Stelzer et al., 2016; Safran et al., 2021; Tolias et al., 2011). This protein appeared to be part of a larger protein–protein interaction network within the two dysregulated proteomes in the dataset. More importantly, multiple studies have drawn a connection between increased RHOA activity and PD-linked phenomena, such as damage of dopaminergic neurons or upregulation of alpha-synuclein (Schmidt S. I. et al., 2022). In addition, an interaction with LRRK2 was discussed, although the results remained inconclusive (Chan et al., 2011). Finally, ACTB represents a ubiquitously expressed protein and it is a crucial component of the cytoskeleton (Stelzer et al., 2016; Safran et al., 2021). As such, it is typically enriched in synapses, where it influences synaptic shape, size, and neurotransmitter release (Gentile et al., 2022). In the cell model, it also appeared among the top 25 interactors within the dysregulated proteome of both EVs and cell lysates and was also part of the MCODE-based functional network. Interestingly, in a G2019S drosophila model and using RNA sequencing, ACTB was identified among the most significant dysregulated gene nodes, suggesting a critical role of LRRK2 in actin cytoskeleton reorganization (Toh et al., 2021).

The other biological process we identified through both GO enrichment analysis and MCODE-based analysis of the shared dysregulated proteome of EVs and cell lysates was RNA processing. The disturbance of RNA processing is being discussed as both a potential cause of neurodegenerative diseases and a target for therapeutic interventions (Nussbacher et al., 2019). RNA-binding proteins were shown to interfere with several steps of RNA metabolism, including splicing, mRNA transport, translation, and degradation. Changes such as dysregulated expression, cellular mislocalization, and aggregation of RNA binding proteins were suggested to result in impaired RNA metabolism in neurodegenerative diseases, although the precise mechanisms of how this leads to neurodegeneration are not fully understood (Nussbacher et al., 2019). Furthermore, we recently showed that LRRK2 mutation carriers display distinct miRNA patterns, connecting LRRK2-driven PD to a disturbed RNA metabolism (Braunger et al., 2024). In this context, the downregulation of AGO1 and its presence among the functional network identified by MOCDE becomes of particular interest. AGO1, which, together with other proteins, forms the RNA-induced silencing complex, binds to microRNAs, and then regulates the translation of mRNAs, thereby playing a crucial role in transcriptional silencing (Stelzer et al., 2016; Safran et al., 2021). In a drosophila model, it was shown that LRRK2 not only associates with AGO1 but also that LRRK2 G2019S negatively regulates AGO1 expression levels (Gehrke et al., 2010). In the cell model, this downregulation seems to be measurable not only in hDaNs but also in EVs, therefore confirming previous study on RNA processing and LRRK2.

Using MCODE, we identified a functional protein–protein interaction network labeled “*Semaphorin interactions*” to be part of the dysregulated proteome. Semaphorins play a role in axonal guidance and neural development but are increasingly linked to neurodegenerative processes (Alto and Terman, 2017; Quintremil et al., 2018). They might be relevant in maintaining the structural and functional integrity of neural circuits, which may be disrupted in neurodegenerative diseases such as PD (Pasterkamp and Giger, 2009). Moreover, specific semaphorins have been implicated in processes such as inflammation and oxidative stress, both of which are critical contributors to the pathology of neurodegenerative diseases (Quintremil et al., 2018; Good et al., 2004). Two proteins within the network were likely the driver for its association with semaphorins. For one, RHOA, which we already discussed above, is likely connected to semaphorin-mediated axonal guidance (Liu and Strittmatter, 2001). Additionally, p21-activated kinase-1 (PAK1) was shown to be suppressed by semaphorin 3A resulting in modulation of inflammation (Huang et al., 2022).

In addition to the above-mentioned proteins SYNJ1, TUBB, RHOA, ACTB, and AGO1, we identified another five proteins from the dataset that were dysregulated both in the cellular and extracellular proteome and appeared as promising biomarker candidates based on probable functional relevance and previous appearances in publications related to PD or LRRK2. Particularly interesting to us was the identification of upregulated levels of sonic hedgehog signaling molecule (SHH) in both EVs and hDaNs. SHH was first described to play an important role in sustaining the chemical and cellular integrity of nigrostriatal circuits over 10 years ago (Gonzalez-Reyes et al., 2012). Since then, using cellular models of both sporadic and familial PD, including LRRK2, we have learned that there is a close interplay of increased SHH activity and ciliary dysfunction (Schmidt S. et al., 2022). Ciliary defects were also shown in LRRK2 mutant mice, including the G2019S variant. Disruption of the SHH signaling pathway, which requires intact cilia, might be a mechanism of neuronal vulnerability in PD patients with LRRK2 mutations, and monitoring extracellular levels of SHH via quantification from EVs might be a promising approach to, for example, monitor treatment efficacies (Khan et al., 2021).

Annexin A1 (ANXA1) is a membrane-bound protein and has anti-inflammatory properties, likely via the inhibition of phospholipase A2 (Stelzer et al., 2016; Safran et al., 2021). Missense variants of ANXA1 were discussed to cause genetic PD, and it was hypothesized that ANXA plays a role in impaired clearance of accumulated alpha-synuclein via microglial defects and neuroinflammation (Darvish et al., 2021). Interestingly, growing evidence suggests a link between LRRK2 and inflammation, in both the periphery and the central nervous system. LRRK2 variants were shown to modulate the risk for Crohn’s disease, a chronic inflammatory bowel disease, and LRRK2 kinase activity was shown to influence microglial activation and pro-inflammatory cytokine production (Hui et al., 2018; Moehle et al., 2012). In the same context, the upregulation of CD44 seems intriguing as this protein is heavily involved in cell–cell interactions, especially in the context of inflammation (Stelzer et al., 2016; Safran et al., 2021). Downregulation of CD44 was shown to decrease microglia-driven neuroinflammation and reduce the loss of dopaminergic neurons in CD44-knockout mice (Wang et al., 2022). A large proteomics study on CSF from both sPD and fPD patients carrying an LRRK2 mutation already revealed upregulated levels of CD44 expression compared to those in healthy controls, therefore being in line with the findings (Karayel et al., 2022).

Galactosylceramidase (GALC) was another intriguing candidate we identified within the dataset. This lysosomal protein represents an important component in glycosphingolipid metabolism and is therefore biochemically closely related to glucocerebrosidase (GBA) (Stelzer et al., 2016; Safran et al., 2021; Senkevich et al., 2023). GBA, in turn, is particularly known for its significance in the context of PD as a multitude of genetic variants modulate the risk of developing the disease (Sidransky et al., 2009). A recent study proposed GALC to also affect the risk of PD and suggested that its dysregulated activity might alter the composition of lysosomal membranes, which due to its involvement in the endolysosomal system links GALC directly to LRRK2 (Senkevich et al., 2023; Erb and Moore, 2020). Additionally, in a small case report, patients with dual mutations in both GBA and LRRK2 displayed increased GALC activity (Usenko et al., 2024). Based on the data, we propose that total protein levels of GALC could also be altered in patient-derived materials, including brain-derived EVs.

Finally, we found dysregulated levels of peroxiredoxin 2 (PRDX2), which is an antioxidant enzyme that contributes to cellular protection against damage from free radical oxygen species (ROS) (Stelzer et al., 2016; Safran et al., 2021). It is well established that regulation of ROS, which physiologically occurs during the mitochondrial electron transfer chain, is essential to cellular homeostasis. Furthermore, several PD-related genes encode proteins tightly involved in the regulation of mitochondrial integrity and oxidative stress, including LRRK2 (Puspita et al., 2017). In this context, PRDX2 overexpression was shown to be protective in a cell model of PD (Liu et al., 2023). Interestingly, it was also shown to be phosphorylated by LRRK2, leading to the loss of dopaminergic neurons (Chua et al., 2020).

### 4.1 Limitations

There are several weaknesses in the study we would like to address. First and foremost, we did not apply biochemical validation experiments to further substantiate the list of biomarker candidates. However, we did find some proteins that had previously been reported to be dysregulated in EVs isolated from larger clinical studies using proteomics and phosphor-proteomics on EVs derived from either CSF or urine and from LRRK2 mutation carriers, such as transketolase (TKT), several members of the peroxiredoxin family (e.g., PRDX3) and the integrin beta family (e.g., ITGB1) (Karayel et al., 2022; Hadisurya et al., 2023). The second weakness relates to the lack of biological replicates. Ideally, additional lines including gene-corrected controls could have been used to potentially increase the applicability of the findings to a broader spectrum of patients. Unfortunately, a very limited number of iPSC lines is available and characterization of LRRK2 expression levels has uncovered significant differences in hDaNs, hampering the usability of the second line. Finally, the approach included a focus on CNS-related GO terms and later the focus on proteins described to be functionally relevant to LRRK2 or PD. We are convinced that this approach allowed for a streamlined analysis and efficient way to identify a comprehensive list of potential biomarker candidates starting from a larger number of proteins. Using Metascape, we have further tried to broaden the approach and single out proteins independent of their CNS-relatedness. However, it is possible that by engaging in a focused approach, we might have overlooked other promising candidates within the dataset.

### 4.2 Future studies

In our view, future studies could build on the discussed findings and use targeted biochemical quantification methods on patient material, such as plasma or CSF-derived EVs, to measure potential differences in expression levels of one or multiple proteins identified throughout this manuscript. To quantify the exact effect LRRK2 activity, which is discussed as the biochemical correlate of LRRK2 mutations, has on the cellular and extracellular proteome, LRRK2 inhibition experiments could be a promising path to follow. With relatively large volumes of CCM needed to reach EV yields great enough to perform proteomics experiments, longer incubation times with LRRK2 inhibitors will be necessary. Therefore, finding the right dosage to treat the cells with over a period of at least 10 days without reducing their viability will be challenging. Finally, using a larger number of cell lines, for example, those available from the FOUNDIN PD consortium, might provide more robust and translatable insights into disease-related changes of the cellular and extracellular proteome in the context of PD-related mutations.

### 4.3 Conclusion

In summary, in the present study, we used iPSC-derived hDaNs and their EVs to characterize the proteomic changes in the context of the LRRK2 G2019S mutation. While previous studies on the proteome of urinary EVs and the CSF of LRRK2 PD patients have been performed, to the best of our knowledge this was the first study that directly characterized the proteome of dopaminergic neurons carrying an LRRK2 mutation and their EVs (Karayel et al., 2022; Hadisurya et al., 2023; Virreira Winter et al., 2021). We identified a plethora of dysregulated proteins and gene ontology enrichment analysis highlighted pathways that are functionally relevant for neurodegeneration. Finally, we focused on proteins that showed dysregulation on both cellular and extracellular levels, intending to increase the robustness of the findings. We thoroughly discussed ten promising protein biomarker candidates, their functional relevance in neurodegeneration and PD as well as their connection to LRRK2, thereby providing researchers with a solid foundation for future biomarker studies.

## Data Availability

The data presented in the study are deposited in the zenodo repository (https://doi.org/10.5281/zenodo.14254138).

## Footnotes

^1^ https://github.com/bigomics/omicsplayground

## Supplementary material

SUPPLEMENTARY FIGURE S1 Statistical analysis of EV marker protein expression. Dots correspond to EVs, triangles correspond to cell lysates. Each data point represents a technical replicate. The y-axis shows the log10 relative abundancy of the respective EV marker protein. In EVs, expression of CD81 was significantly increased compared to cell lysates **(A)**. Data corresponding to L1 GC are depicted as round dots while triangles represent data from L1 G2019S. Here, expression of CD81 in EVs derived from L1 G2019S seemed to significantly higher compared to L1 GC. In contrast, the other EV markers were not significantly different between the lines.

SUPPLEMENTARY Figure S2 Cryo-TEM images of vesicles isolated from hDaNs. Images were taken from vesicles isolated from L1 GC **(A, B)** as well as from L1 G2019S **(C, D)**. Vesicles appeared as solitary, spherical and membrane-encapsulated structures. No morphological difference between genotypes were observed.

SUPPLEMENTARY FIGURE S3 Literature review for dysregulated proteins. For 22 of the 123 proteins dysregulated in both EVs and hDaNs, there was at least one publication connecting it to both Parkinson’s disease **(A)** and LRRK2 **(B)**.
