# Supplementary figures and images for: The cellular and extracellular proteomic signature of human dopaminergic neurons carrying the LRRK2 G2019S mutation
Source: Front Neurosci. 2024 Dec 12;18:1502246. doi: 10.3389/fnins.2024.1502246 (PMC11669673; doi:10.3389/fnins.2024.1502246)

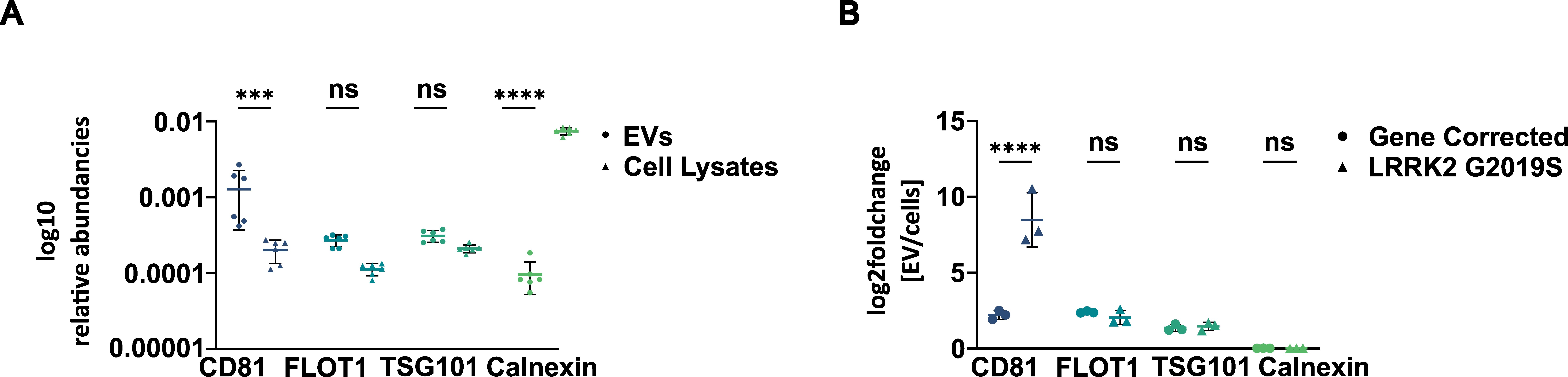

Supplement: SUPPLEMENTARY FIGURE S1 — Statistical analysis of EV marker protein expression. Dots correspond to EVs, triangles correspond to cell lysates. Each data point represents a technical replicate. The y-axis shows the log10 relative abundancy of the respective EV marker protein. In EVs, expression of CD81 was significantly increased compared to cell lysates (A). Data corresponding to L1 GC are depicted as round dots while triangles represent data from L1 G2019S. Here, expression of CD81 in EVs derived from L1 G2019S seemed to significantly higher compared to L1 GC. In contrast, the other EV markers were not significantly different between the lines. [file Image_1.JPEG]

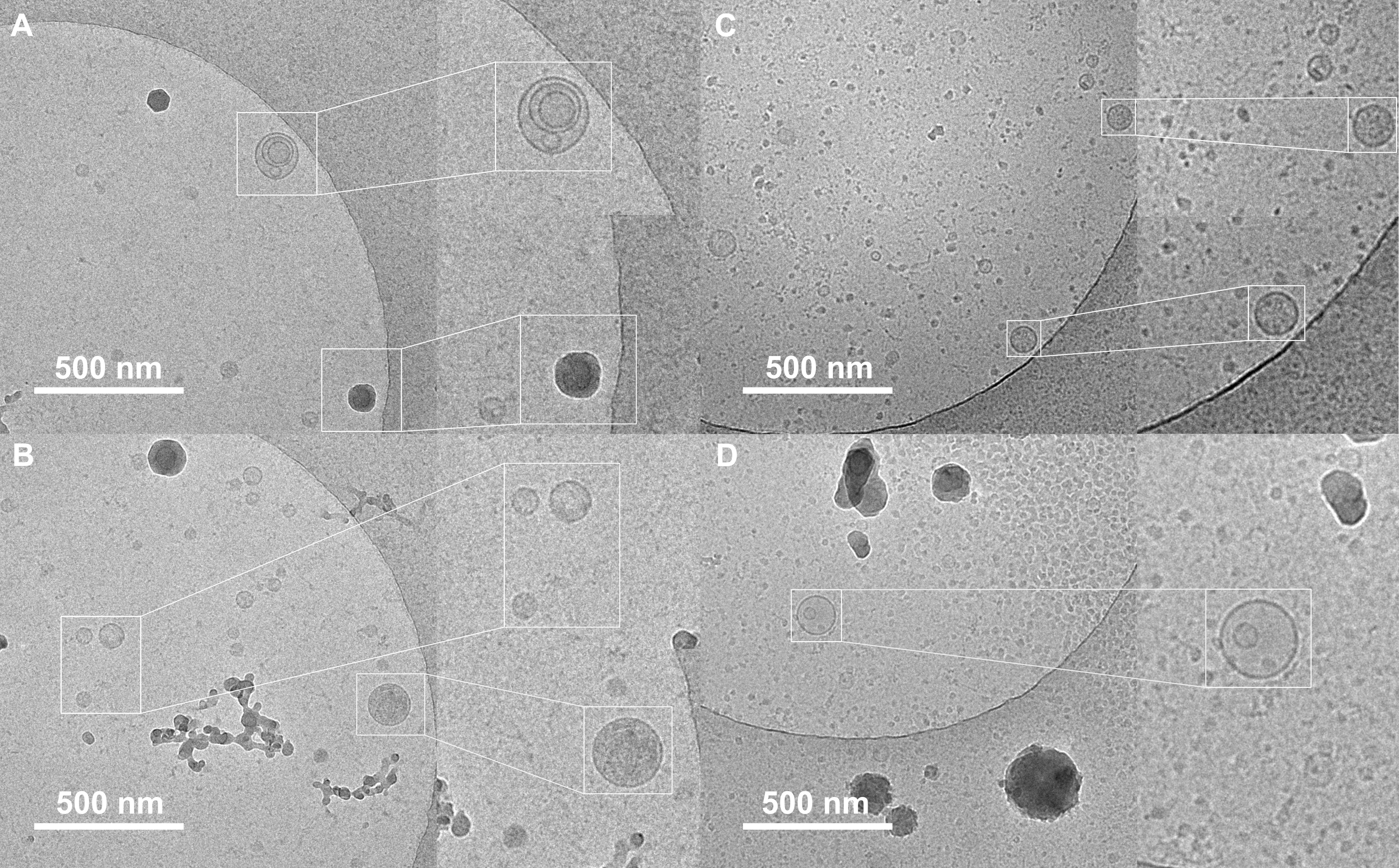

Supplement: SUPPLEMENTARY Figure S2 — Cryo-TEM images of vesicles isolated from hDaNs. Images were taken from vesicles isolated from L1 GC (A, B) as well as from L1 G2019S (C, D). Vesicles appeared as solitary, spherical and membrane-encapsulated structures. No morphological difference between genotypes were observed. [file Image_2.JPEG]

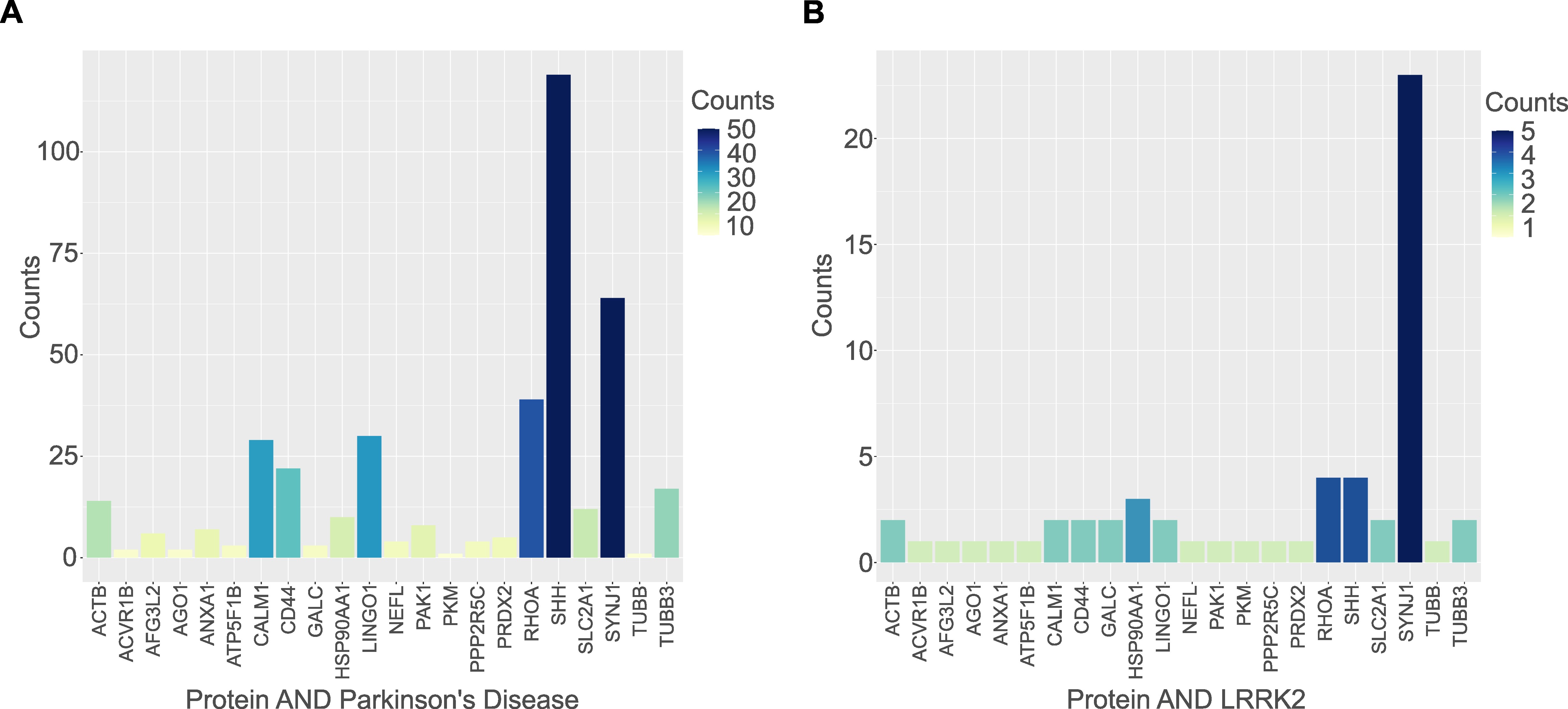

Supplement: SUPPLEMENTARY FIGURE S3 — Literature review for dysregulated proteins. For 22 of the 123 proteins dysregulated in both EVs and hDaNs, there was at least one publication connecting it to both Parkinson’s disease (A) and LRRK2 (B). [file Image_3.JPEG]
