# Supplementary material for: The cellular and extracellular proteomic signature of human dopaminergic neurons carrying the LRRK2 G2019S mutation
Source: Front Neurosci. 2024 Dec 12;18:1502246. doi: 10.3389/fnins.2024.1502246 (PMC11669673; doi:10.3389/fnins.2024.1502246)
Supplement: Supplementary file 4 [file Table_1.DOCX]

| **Name** | **Ingredient** | **Quantity** | **Order Number** |
| --- | --- | --- | --- |
| **50:50 media** | Neurobasal media | 50% | Thermo Fisher Scientific, #21103-049 |
|  | DMEM/F12 | 50% | Thermo Fisher Scientific, #11-330-057 |
| **Base media**  **(= 50:50 media +)** | Penicillin/Streptomycin | 1% | Merck, #A2213 |
|  | GlutaMax | 1% | Thermo Fisher Scientific, #35050-038 |
|  | B27 supplement | 1% | Thermo Fisher Scientific, #12587-010 |
|  | N2 supplement | 0.5% | Thermo Fisher Scientific, #17502-048 |
| **NPC media**  **(= Base media +)** | PMA | 0.5 µM | Merck, #540220 |
|  | CHIR | 3 µM | Axon Medchem, #Axon1386 |
|  | Ascorbic acid | 200 µM | Sigma-Aldrich, #A4544 |
| **D7 media**  **(= Base media +)** | BDNF | 20 ng/ml | PeproTech, #450-02 |
|  | FGF8 | 10 ng/ml | PeproTech, #100-25 |
|  | PMA | 1 µM | Merck, #540220 |
|  | Ascorbic acid | 200 µM | Sigma-Aldrich, #A4544 |
| **Maturation media**  **(= Base media +)** | BDNF | 10 ng/ml | PeproTech, #450-02 |
|  | GDNF | 10 ng/ml | PeproTech, #450-10 |
|  | TGF-β3 | 1 ng/ml | PeproTech, #AF-100-36E |
|  | ascorbic acid | 200 µM | Sigma-Aldrich, #A4544 |
|  | dbcAMP | 500 µM | PanReac AppliChem, #A0455 |
|  | DAPT | 10 µM | Selleckchem, #S2215 |

Supplemental Table S1: Overview of cell culture media.
