# Supplementary material for: The cellular and extracellular proteomic signature of human dopaminergic neurons carrying the LRRK2 G2019S mutation
Source: Front Neurosci. 2024 Dec 12;18:1502246. doi: 10.3389/fnins.2024.1502246 (PMC11669673; doi:10.3389/fnins.2024.1502246)
Supplement: Supplementary file 5 [file Table_2.DOCX]

Supplemental Table S2: CNS related terms used for filtering significant GO terms.

| **CNS related terms used for filtering** | "brain", "midbrain", "nervous system", "neuron", "substantia nigra", "synapse",  "central nervous system", "brain development", "brain stem", "neurogenesis",  "neurodegeneration", "dopamine", "dopaminergic neuron", "alpha-synuclein", "neuroinflammation", "inflammation", "neuroprotection", "tau protein", "mitochondria dysfunction", "oxidative stress", "neurotransmitter", "protein aggregation", "apoptosis", "autophagy", "neuroplasticity", "plasticity", "blood-brain barrier", "gliosis", "astrocyte", "microglia", "oligodendrocyte", "myelin", "white matter", "grey matter", "cerebrospinal fluid" |
| --- | --- |
