# Supplementary material for: The cellular and extracellular proteomic signature of human dopaminergic neurons carrying the LRRK2 G2019S mutation
Source: Front Neurosci. 2024 Dec 12;18:1502246. doi: 10.3389/fnins.2024.1502246 (PMC11669673; doi:10.3389/fnins.2024.1502246)
Supplement: Supplementary file 6 [file Table_3.DOCX]

Supplemental Table S3. GO enrichment analysis for cellular compartment of common EV proteome.

| **GO:ID** | **description** | **adjusted**  **p-value** | **protein count** | **names** |
| --- | --- | --- | --- | --- |
| GO:0072562 | cell membrane microparticle | 1.84E-18 | 17 | HPX,ALB,TF,ITIH4,LGALS3BP,FN1,ACTB,HSPA8,IGKC,IGHG4,IGHG2,SDCBP,IGHG3,CLU,KRT1,CFB,IGLC2 |
| GO:0062023 | collagen-containing extracellular matrix | 1.39E-14 | 20 | HPX,SPON1,TNC,ITIH4,LGALS3BP,FN1,GPC2,L1CAM,PKM,PXDN,AGRN,THBS3,COL6A1,APLP1,CLU,HSP90AA1,KRT1,VCAN,MMP2,LAMB2 |
| GO:0034774 | secretory granule lumen | 7.34E-12 | 16 | ALB,TF,ITIH4,LGALS3BP,FN1,HSPA8,TUBB,PKM,SDCBP,APP,CLU,HSP90AA1,CAND1,VCP,EEF2,SPTAN1 |
| GO:0060205 | cytoplasmic vesicle lumen | 7.34E-12 | 16 | ALB,TF,ITIH4,LGALS3BP,FN1,HSPA8,TUBB,PKM,SDCBP,APP,CLU,HSP90AA1,CAND1,VCP,EEF2,SPTAN1 |
| GO:0031983 | vesicle lumen | 7.34E-12 | 16 | ALB,TF,ITIH4,LGALS3BP,FN1,HSPA8,TUBB,PKM,SDCBP,APP,CLU,HSP90AA1,CAND1,VCP,EEF2,SPTAN1 |
| GO:0005775 | vacuolar lumen | 1.29E-06 | 9 | GPC2,HSPA8,TUBB,AGRN,SDCBP,HSP90AA1,NCAN,VCAN,VCP |
| GO:0071735 | IgG immunoglobulin complex | 1.67E-06 | 4 | IGKC,IGHG4,IGHG2,IGHG3 |
| GO:0150034 | distal axon | 4.23E-06 | 10 | ACTB,HSPA8,L1CAM,TUBB3,DCC,APP,MAP1B,CRMP1,HSP90AA1,OLFM1 |
| GO:0030426 | growth cone | 8.70E-06 | 8 | L1CAM,TUBB3,DCC,APP,MAP1B,CRMP1,HSP90AA1,OLFM1 |
| GO:0030427 | site of polarized growth | 9.24E-06 | 8 | L1CAM,TUBB3,DCC,APP,MAP1B,CRMP1,HSP90AA1,OLFM1 |
| GO:0005788 | endoplasmic reticulum lumen | 9.24E-06 | 10 | ALB,TF,SPON1,TNC,FN1,COL6A1,APP,CLU,VCAN,LAMB2 |
| GO:0043025 | neuronal cell body | 1.14E-05 | 12 | CNTN2,L1CAM,TUBB3,APP,CKB,DPYSL5,MAP1B,CRMP1,SYT11,HSP90AA1,PTPRF,OLFM1 |
| GO:1904813 | ficolin-1-rich granule lumen | 1.14E-05 | 7 | HSPA8,PKM,HSP90AA1,CAND1,KRT1,VCP,EEF2 |
| GO:0005604 | basement membrane | 1.78E-05 | 6 | TNC,FN1,PXDN,AGRN,APLP1,LAMB2 |
| GO:0044295 | axonal growth cone | 3.78E-05 | 4 | L1CAM,DCC,HSP90AA1,OLFM1 |
| GO:0043202 | lysosomal lumen | 3.86E-05 | 6 | GPC2,HSPA8,AGRN,HSP90AA1,NCAN,VCAN |
| GO:0005796 | Golgi lumen | 5.74E-05 | 6 | MUC5AC,GPC2,AGRN,APP,NCAN,VCAN |
| GO:0101002 | ficolin-1-rich granule | 0.00011851 | 7 | HSPA8,PKM,HSP90AA1,CAND1,KRT1,VCP,EEF2 |
| GO:0031091 | platelet alpha granule | 0.00039814 | 5 | ALB,FN1,APP,CLU,VPS33B |
| GO:0031093 | platelet alpha granule lumen | 0.001798 | 4 | ALB,FN1,APP,CLU |
| GO:0005925 | focal adhesion | 0.00307742 | 8 | TNC,ACTB,HSPA8,L1CAM,VIM,SDCBP,AHNAK,LRP1 |
| GO:0030055 | cell-substrate junction | 0.00343835 | 8 | TNC,ACTB,HSPA8,L1CAM,VIM,SDCBP,AHNAK,LRP1 |
| GO:0043204 | perikaryon | 0.00418272 | 5 | APP,MAP1B,CRMP1,SYT11,OLFM1 |
| GO:0030139 | endocytic vesicle | 0.00460979 | 7 | HPX,TF,VIM,SYT11,HSP90AA1,RPS27A,LRP1 |
| GO:0099572 | postsynaptic specialization | 0.00588133 | 7 | HSPA8,CLSTN1,CLSTN3,DCC,SDCBP,MAP1B,SYT11 |
| GO:0005641 | nuclear envelope lumen | 0.0065904 | 2 | TUBB,APP |
| GO:0019814 | immunoglobulin complex | 0.0082426 | 4 | IGKC,IGHG4,IGHG2,IGHG3 |
| GO:0055037 | recycling endosome | 0.01053687 | 5 | TF,TUBA1A,APP,SYT11,VPS33B |
| GO:0005765 | lysosomal membrane | 0.01053687 | 7 | HSPA8,UBA1,COL6A1,SYT11,VPS33B,AHNAK,LRP1 |
| GO:0098852 | lytic vacuole membrane | 0.01053687 | 7 | HSPA8,UBA1,COL6A1,SYT11,VPS33B,AHNAK,LRP1 |
| GO:0031089 | platelet dense granule lumen | 0.01106389 | 2 | ITIH4,LGALS3BP |
| GO:0097440 | apical dendrite | 0.01233551 | 2 | MAP1B,CLU |
| GO:0098637 | protein complex involved in cell-matrix adhesion | 0.01539659 | 2 | TNC,LAMB2 |
| GO:0044304 | main axon | 0.01539659 | 3 | CNTN2,APP,MAP1B |
| GO:0014069 | postsynaptic density | 0.01552222 | 6 | CLSTN1,CLSTN3,DCC,SDCBP,MAP1B,SYT11 |
| GO:0005774 | vacuolar membrane | 0.01552222 | 7 | HSPA8,UBA1,COL6A1,SYT11,VPS33B,AHNAK,LRP1 |
| GO:0005874 | microtubule | 0.01552222 | 7 | TUBA4A,TUBA1A,TUBB,DPYSL2,TUBB3,MAP1B,TUBB2A |
| GO:0032279 | asymmetric synapse | 0.01800821 | 6 | CLSTN1,CLSTN3,DCC,SDCBP,MAP1B,SYT11 |
| GO:0005905 | clathrin-coated pit | 0.01896262 | 3 | TF,APP,LRP1 |
| GO:0031594 | neuromuscular junction | 0.01896262 | 3 | TUBA1A,APP,LAMB2 |
| GO:0042827 | platelet dense granule | 0.01896262 | 2 | ITIH4,LGALS3BP |
| GO:0071682 | endocytic vesicle lumen | 0.02218801 | 2 | HPX,HSP90AA1 |
| GO:0098984 | neuron to neuron synapse | 0.02344329 | 6 | CLSTN1,CLSTN3,DCC,SDCBP,MAP1B,SYT11 |
| GO:0005791 | rough endoplasmic reticulum | 0.02403826 | 3 | PKM,UBA1,APP |
| GO:0097060 | synaptic membrane | 0.02476899 | 6 | HSPA8,CNTN2,CLSTN1,CLSTN3,DCC,SYT11 |
| GO:0045211 | postsynaptic membrane | 0.02627288 | 5 | HSPA8,CNTN2,CLSTN1,CLSTN3,DCC |
| GO:0045171 | intercellular bridge | 0.02829524 | 3 | TUBB,TUBB3,TUBB2A |
| GO:0035578 | azurophil granule lumen | 0.02947214 | 3 | TUBB,SDCBP,VCP |
| GO:0030135 | coated vesicle | 0.04037916 | 5 | TF,HSPA8,APP,SYT11,VPS33B |
| GO:0042470 | melanosome | 0.04906456 | 3 | HSPA8,SDCBP,HSP90AA1 |
| GO:0048770 | pigment granule | 0.04906456 | 3 | HSPA8,SDCBP,HSP90AA1 |
