# Supplementary material for: The cellular and extracellular proteomic signature of human dopaminergic neurons carrying the LRRK2 G2019S mutation
Source: Front Neurosci. 2024 Dec 12;18:1502246. doi: 10.3389/fnins.2024.1502246 (PMC11669673; doi:10.3389/fnins.2024.1502246)
Supplement: Supplementary file 7 [file Table_4.DOCX]

Supplemental Table S4. GO enrichment analysis for CNS related biological processes of the EV proteome upregulated in L1 G2019S.

| **GO:ID** | **description** | **adjusted**  **p-value** | **protein count** | **names** |
| --- | --- | --- | --- | --- |
| GO:0050808 | synapse organization | 6.92e-05 | 22 | DAG1,PCDHB2,PTPRF,ITGB1,SDCBP,RAB39B,LAMB2,PCDHB16,DIP2A,GRID2,AGRN,AFG3L2,RHOA,THBS2,PCDHB14,TNC,PCDHGC4,SLIT1,RAP2A,DNER,NTNG2,ARF4 |
| GO:0007416 | synapse assembly | 0.0036246 | 11 | PCDHB2,SDCBP,PCDHB16,GRID2,AGRN,THBS2,PCDHB14,SLIT1,RAP2A,DNER,NTNG2 |
| GO:0031102 | neuron projection regeneration | 0.00484373 | 6 | DAG1,PTPRF,LAMB2,TNC,MTR,APOD |
| GO:0099072 | regulation of postsynaptic membrane neurotransmitter receptor levels | 0.02435964 | 6 | DAG1,AP2M1,AP2A1,RAP2A,CALY,STX3 |
| GO:0050807 | regulation of synapse organization | 0.027474344 | 10 | DAG1,ITGB1,GRID2,AGRN,RHOA,THBS2,SLIT1,RAP2A,NTNG2,ARF4 |
| GO:0050803 | regulation of synapse structure or activity | 0.031031203 | 10 | DAG1,ITGB1,GRID2,AGRN,RHOA,THBS2,SLIT1,RAP2A,NTNG2,ARF4 |
| GO:1990138 | neuron projection extension | 0.040325876 | 8 | ITGB1,SEMA6D,LAMB2,FN1,SYT1,DDR1,SLIT1,ALCAM |
| GO:0045665 | negative regulation of neuron differentiation | 0.043691617 | 5 | ITGB1,JAG1,SHH,RHOA,CNTN4 |
| GO:0098884 | postsynaptic neurotransmitter receptor internalization | 0.047513767 | 3 | AP2M1,AP2A1,CALY |
