# Supplementary material for: The cellular and extracellular proteomic signature of human dopaminergic neurons carrying the LRRK2 G2019S mutation
Source: Front Neurosci. 2024 Dec 12;18:1502246. doi: 10.3389/fnins.2024.1502246 (PMC11669673; doi:10.3389/fnins.2024.1502246)
Supplement: Supplementary file 8 [file Table_5.DOCX]

Supplemental Table S5. GO enrichment analysis for CNS related biological processes of the EV proteome downregulated in L1 G2019S.

| **GO:ID** | **description** | **adjusted**  **p-value** | **protein count** | **names** |
| --- | --- | --- | --- | --- |
| GO:0098962 | regulation of postsynaptic neurotransmitter receptor activity | 0.0188626 | 4 | NRXN2/DLGAP4/AKAP9/NPTX1 |
| GO:0050808 | synapse organization | 0.02442992 | 15 | STAU1/CAPRIN1/EPHB1/FLNA/PCDHB16/NRXN2/CDH10/ACTB/RELN/NEFL/CLSTN2/NPTX1/TUBB/LRFN3/CHD4 |
