# Supplementary material for: The cellular and extracellular proteomic signature of human dopaminergic neurons carrying the LRRK2 G2019S mutation
Source: Front Neurosci. 2024 Dec 12;18:1502246. doi: 10.3389/fnins.2024.1502246 (PMC11669673; doi:10.3389/fnins.2024.1502246)
Supplement: Supplementary file 9 [file Table_6.DOCX]

Supplemental Table S6. GO enrichment analysis for CNS related biological processes of the cellular proteome upregulated in L1 G2019S hDaNs.

| **GO:ID** | **description** | **adjusted**  **p-value** | **protein**  **count** | **names** |
| --- | --- | --- | --- | --- |
| GO:0099003 | vesicle-mediated transport in synapse | 1.82E-14 | 46 | ACTB,SYP,RAB3B,ATP6V1G1,CTBP1,SYT1,RAP1B,ARF6,BSN,DNM1,BRSK1,BRAF,ATP6V0A1,AP3M2,SLC18A2,SH3GL1,SYT12,RALA,FMR1,DGKQ,DDC,PREPL,CPLX4,SNCG,SV2A,CPLX1,SYNJ1,VPS35,SEPTIN5,CALY,TH,SNCB,CTNNB1,STX1A,SNCA,RAP1A,CADPS,AMPH,RAB5A,DNAJC6,STX1B,AP2A1,CTBP2,DNM2,PRRT2,STXBP1 |
| GO:0001505 | regulation of neurotransmitter levels | 2.09E-13 | 43 | SYP,NRXN2,RAB3B,SYT1,RAP1B,SYNGR3,BRSK1,BRAF,SLC18A2,CLN8,SYT12,LIN7A,MAOA,MAOB,FMR1,GAD1,PREPL,COMT,GCHFR,CPLX4,SNCG,SV2A,LIN7B,CPLX1,LIN7C,ALDH5A1,SYNJ1,SEPTIN5,CAMK2A,PTPRN2,TH,STX1A,SNCA,RAP1A,SLC1A3,CADPS,RAB5A,AGTPBP1,STX1B,CTBP2,ASIC1,PRRT2,STXBP1 |
| GO:0006836 | neurotransmitter transport | 3.95E-10 | 37 | SYP,NRXN2,RAB3B,SYT1,RAP1B,SYNGR3,BRSK1,BRAF,SLC18A2,CLN8,SYT12,LIN7A,FMR1,DDC,PREPL,CPLX4,SNCG,SV2A,LIN7B,CPLX1,LIN7C,SYNJ1,SEPTIN5,CAMK2A,PTPRN2,TH,STX1A,SNCA,RAP1A,SLC1A3,CADPS,RAB5A,STX1B,CTBP2,ASIC1,PRRT2,STXBP1 |
| GO:0007269 | neurotransmitter secretion | 9.10E-10 | 30 | SYP,NRXN2,SYT1,RAP1B,BRSK1,BRAF,SYT12,LIN7A,FMR1,PREPL,CPLX4,SNCG,SV2A,LIN7B,CPLX1,LIN7C,SYNJ1,SEPTIN5,CAMK2A,PTPRN2,STX1A,SNCA,RAP1A,CADPS,RAB5A,STX1B,CTBP2,ASIC1,PRRT2,STXBP1 |
| GO:0099643 | signal release from synapse | 9.10E-10 | 30 | SYP,NRXN2,SYT1,RAP1B,BRSK1,BRAF,SYT12,LIN7A,FMR1,PREPL,CPLX4,SNCG,SV2A,LIN7B,CPLX1,LIN7C,SYNJ1,SEPTIN5,CAMK2A,PTPRN2,STX1A,SNCA,RAP1A,CADPS,RAB5A,STX1B,CTBP2,ASIC1,PRRT2,STXBP1 |
| GO:0010975 | regulation of neuron projection development | 2.69E-09 | 57 | CRMP1,PAK2,ARF6,PLXNA1,ROBO2,RAP2A,FYN,BRSK1,PPP3CA,MARK1,CRTC1,BRAF,PLXNC1,KIAA0319,MAP2K1,PTPN9,GORASP1,ALK,SYNGAP1,RAPGEF1,VLDLR,NCK1,NEGR1,FIG4,ARHGAP33,DPYSL3,RHOA,PAK1,MAP6,PAFAH1B1,STMN2,CFL1,VIM,PLXNA4,PTPRG,SPOCK1,SEMA4C,CNTN1,NRCAM,RAP1A,SHTN1,GDI1,CARM1,MAPT,NEFL,PLXNA3,INPP5F,CAMK2B,STX1B,NEDD4,EFNB3,SERPINI1,ARSB,RUFY3,PAK3,DPYSL5,RTN4 |
| GO:0050808 | synapse organization | 4.91E-07 | 53 | ACTB,NRXN2,RAP1B,ARF6,INA,EGLN1,ROBO2,RAP2A,BSN,FYN,GPM6A,TUBB,PPFIA1,EIF4G1,NPTX1,PLXNC1,AFG3L2,DLG5,DIP2A,CTNND2,SYNGAP1,PCDHB2,NEGR1,ARHGAP33,SDCBP,SLC25A46,RHOA,SNCG,FARP1,PAFAH1B1,RAC3,CFL1,ACTN1,VPS35,PLXNA4,SNCB,CTNNB1,SNCA,NRCAM,ARF1,HOMER1,MAPT,NEFL,EPHB1,CAMK2B,NEDD4,ADD2,EFNB3,GRID1,CTBP2,PAK3,CACNB1,RHOB |
| GO:0051588 | regulation of neurotransmitter transport | 3.62E-06 | 20 | SYP,RAB3B,SYT1,RAP1B,SYNGR3,BRAF,FMR1,PREPL,CPLX4,SNCG,CPLX1,SEPTIN5,CAMK2A,STX1A,SNCA,RAP1A,RAB5A,STX1B,ASIC1,STXBP1 |
| GO:0046928 | regulation of neurotransmitter secretion | 4.27E-06 | 18 | SYP,SYT1,RAP1B,BRAF,FMR1,PREPL,CPLX4,SNCG,CPLX1,SEPTIN5,CAMK2A,STX1A,SNCA,RAP1A,RAB5A,STX1B,ASIC1,STXBP1 |
| GO:0048167 | regulation of synaptic plasticity | 1.70E-05 | 29 | SYP,AKAP5,BRSK1,MPP2,SCGN,CRTC1,BRAF,HRAS,SLC4A10,SYT12,SYNGAP1,GRIK2,MAP1A,FMR1,SORCS3,GRIN2C,CAMK2A,CALB2,GIPC1,SNCA,GRIA1,ARF1,MAPK1,MAPT,RAB5A,CAMK2B,NCDN,PRRT2,STXBP1 |
| GO:0050803 | regulation of synapse structure or activity | 3.08E-05 | 31 | ARF6,EGLN1,ROBO2,RAP2A,FYN,GPM6A,TUBB,EIF4G1,PLXNC1,DLG5,SYNGAP1,NEGR1,ARHGAP33,RHOA,FARP1,PAFAH1B1,CFL1,VPS35,CTNNB1,SNCA,NRCAM,HOMER1,NEFL,EPHB1,CAMK2B,STX1B,NEDD4,EFNB3,GRID1,PAK3,RHOB |
| GO:0035418 | protein localization to synapse | 5.34E-05 | 16 | NRXN2,BSN,NPTX1,HRAS,MAPK8IP3,RAB11A,MAP1A,GRIN2C,CPLX1,HSPB1,VPS35,KIF5A,RAP1A,HOMER1,MAPT,STX1B |
| GO:0021762 | substantia nigra development | 5.58E-05 | 12 | ACTB,BASP1,CALM1,INA,SYNGR3,DYNLL1,MAOB,GNB4,RHOA,CNP,YWHAE,G6PD |
| GO:0030901 | midbrain development | 8.29E-05 | 17 | ACTB,BASP1,CALM1,INA,SYNGR3,DYNLL1,DLG5,SHH,MAOB,GNB4,RHOA,CSNK1E,CNP,WLS,CTNNB1,YWHAE,G6PD |
| GO:0016236 | macroautophagy | 9.54E-05 | 36 | ATP6V1G1,CALM1,LRBA,SLC25A4,RALB,STAM2,ATP6V0A1,MAP3K7,CHMP3,RETREG1,VPS26A,VPS41,SNX5,EI24,SPTLC1,RAB19,STX17,VPS39,RAB23,PIP4K2B,VPS35,RAB33B,C9orf72,SH3GLB1,EXOC7,TEX264,EXOC1,TSC2,RAB5A,RAB1A,NEDD4,SLC25A5,GBA1,VDAC1,LRSAM1,EXOC4 |
| GO:0050807 | regulation of synapse organization | 0.000122815 | 29 | ARF6,EGLN1,ROBO2,RAP2A,FYN,GPM6A,TUBB,EIF4G1,PLXNC1,DLG5,NEGR1,ARHGAP33,RHOA,FARP1,PAFAH1B1,CFL1,VPS35,CTNNB1,SNCA,NRCAM,HOMER1,NEFL,EPHB1,CAMK2B,NEDD4,EFNB3,GRID1,PAK3,RHOB |
| GO:0099173 | postsynapse organization | 0.000131784 | 25 | ACTB,NRXN2,ARF6,INA,FYN,NPTX1,DLG5,DIP2A,CTNND2,SYNGAP1,ARHGAP33,FARP1,PAFAH1B1,RAC3,CFL1,ACTN1,VPS35,NRCAM,ARF1,HOMER1,NEFL,EPHB1,CAMK2B,GRID1,PAK3 |
| GO:0010977 | negative regulation of neuron projection development | 0.00020559 | 21 | CRMP1,ARF6,KIAA0319,PTPN9,SYNGAP1,DPYSL3,RHOA,PAFAH1B1,STMN2,VIM,PTPRG,SPOCK1,SEMA4C,GDI1,CARM1,PLXNA3,INPP5F,STX1B,EFNB3,RUFY3,RTN4 |
| GO:0099072 | regulation of postsynaptic membrane neurotransmitter receptor levels | 0.000611844 | 15 | RAP2A,PPFIA1,NPTX1,HRAS,ERBIN,PORCN,HSP90AA1,RAB11A,RALA,CPLX1,VPS35,CALY,RAP1A,STX1B,AP2A1 |
| GO:0016241 | regulation of macroautophagy | 0.000718018 | 21 | ATP6V1G1,SLC25A4,RALB,ATP6V0A1,MAP3K7,VPS26A,SNX5,SPTLC1,PIP4K2B,VPS35,C9orf72,SH3GLB1,EXOC7,EXOC1,TSC2,NEDD4,SLC25A5,GBA1,VDAC1,LRSAM1,EXOC4 |
| GO:1990138 | neuron projection extension | 0.00074308 | 22 | DCLK1,SYT1,PLXNA1,KIAA0319,HSP90AA1,SYT17,CPNE5,PAFAH1B1,PLXNA4,C9orf72,CTNNB1,SEMA4C,NRCAM,SHTN1,GDI1,MAPT,PLXNA3,RUFY3,DNM2,MUL1,RAPH1,RTN4 |
| GO:0042133 | neurotransmitter metabolic process | 0.000960551 | 8 | MAOA,MAOB,GAD1,COMT,GCHFR,ALDH5A1,TH,AGTPBP1 |
| GO:0048168 | regulation of neuronal synaptic plasticity | 0.002006493 | 11 | SYP,HRAS,SLC4A10,SYNGAP1,GRIK2,GRIN2C,CAMK2A,SNCA,RAB5A,CAMK2B,NCDN |
| GO:0048169 | regulation of long-term neuronal synaptic plasticity | 0.002198018 | 7 | SYP,HRAS,SYNGAP1,GRIK2,SNCA,RAB5A,CAMK2B |
| GO:0106027 | neuron projection organization | 0.002390022 | 14 | FYN,PLS1,DIP2A,CTNND2,MAP1A,ARHGAP33,PAFAH1B1,CFL1,VPS35,ARF1,HOMER1,EPHB1,CAMK2B,PAK3 |
| GO:0001764 | neuron migration | 0.002482525 | 21 | DCLK1,PLXNA1,FYN,GPM6A,MARK1,KIAA0319,HSP90AA1,ASTN1,NAV1,RHOA,ZMIZ1,PAFAH1B1,CAMK2A,SPOCK1,CTNNB1,NRCAM,SHTN1,PLXNA3,CAMK2B,YWHAE,DCX |
| GO:0042417 | dopamine metabolic process | 0.002981419 | 9 | GCH1,MAOA,MAOB,DDC,COMT,VPS35,TH,SNCB,SNCA |
| GO:0042416 | dopamine biosynthetic process | 0.004075262 | 5 | GCH1,DDC,VPS35,TH,SNCA |
| GO:0021954 | central nervous system neuron development | 0.00468145 | 13 | DCLK1,PLXNA1,SLC4A10,HSP90AA1,ZMIZ1,PAFAH1B1,RAC3,PLXNA4,MAPT,PLXNA3,EPHB1,AGTPBP1,DCLK2 |
| GO:0050767 | regulation of neurogenesis | 0.005729758 | 34 | PLXNA1,ROBO2,PPP3CA,BRAF,PLXNC1,KIAA0319,MAP2K1,GORASP1,SHH,SYNGAP1,SLC7A5,HMGB2,TENM4,CTNNA1,KIFAP3,MAP6,PAFAH1B1,YTHDF2,SYNJ1,PLXNA4,CTNNB1,SEMA4C,SHTN1,GDI1,MAPT,NEFL,PLXNA3,CAMK2B,EFNB3,LRP2,RUFY3,PAK3,DPYSL5,RTN4 |
| GO:0051960 | regulation of nervous system development | 0.006025145 | 39 | PLXNA1,ROBO2,PPP3CA,BRAF,PLXNC1,DLG5,KIAA0319,MAP2K1,GORASP1,SHH,SYNGAP1,SLC7A5,FIG4,HMGB2,TENM4,CTNNA1,KIFAP3,MAP6,PAFAH1B1,YTHDF2,SYNJ1,PLXNA4,CTSC,CTNNB1,SEMA4C,SHTN1,GDI1,MAPT,NEFL,TPPP,PLXNA3,EPHB1,CAMK2B,EFNB3,LRP2,RUFY3,PAK3,DPYSL5,RTN4 |
| GO:0051590 | positive regulation of neurotransmitter transport | 0.0074739 | 6 | RAB3B,SYT1,STX1A,SNCA,STX1B,STXBP1 |
| GO:0014046 | dopamine secretion | 0.0074739 | 8 | SYT1,SLC18A2,SYT12,SYT17,SYT13,SNCG,GABBR1,SNCA |
| GO:0021859 | pyramidal neuron differentiation | 0.0074739 | 5 | SLC4A10,ZMIZ1,PLXNA3,DCLK2,GBA1 |
| GO:0007416 | synapse assembly | 0.007901242 | 21 | NRXN2,ARF6,ROBO2,RAP2A,BSN,GPM6A,EIF4G1,NPTX1,DLG5,PCDHB2,NEGR1,SDCBP,SLC25A46,FARP1,VPS35,CTNNB1,SNCA,NRCAM,EPHB1,ADD2,EFNB3 |
| GO:0010976 | positive regulation of neuron projection development | 0.008145843 | 18 | FYN,CRTC1,BRAF,ALK,RAPGEF1,VLDLR,NCK1,NEGR1,FIG4,DPYSL3,PAFAH1B1,STMN2,CNTN1,RAP1A,CAMK2B,SERPINI1,ARSB,PAK3 |
| GO:0010506 | regulation of autophagy | 0.008680553 | 31 | ATP6V1G1,CISD1,SLC25A4,EIF4G1,RALB,ATP6V0A1,MAP3K7,KLHL22,FOXK1,VPS26A,SNX5,SPTLC1,SLC7A5,HSPB1,PIP4K2B,VPS35,DEPDC5,C9orf72,SNCA,SH3GLB1,EXOC7,PIK3R2,EXOC1,TSC2,MAPT,NEDD4,SLC25A5,GBA1,VDAC1,LRSAM1,EXOC4 |
| GO:0001956 | positive regulation of neurotransmitter secretion | 0.009394202 | 5 | SYT1,STX1A,SNCA,STX1B,STXBP1 |
| GO:0097120 | receptor localization to synapse | 0.009697991 | 10 | NPTX1,HRAS,RAB11A,STX7,CPLX1,KIF3B,VPS35,KIF5A,RAP1A,STX1B |
| GO:0015872 | dopamine transport | 0.010431708 | 9 | RAB3B,SYT1,SLC18A2,SYT12,SYT17,SYT13,SNCG,GABBR1,SNCA |
| GO:0032288 | myelin assembly | 0.010454042 | 6 | FIG4,ANK2,TENM4,EPB41L3,CNTN1,TPPP |
| GO:0070997 | neuron death | 0.01216517 | 32 | EGLN1,FYN,NQO1,EIF4G1,BRAF,HRAS,NCOA7,CLN8,RETREG1,SYNGAP1,GRIK2,CHP1,CD200,SCN2A,RHOA,SNCG,DIABLO,FADD,VPS35,SNCB,CTNNB1,SNCA,CORO1A,MAPT,NEFL,EPHB1,GCLC,GBA1,G6PD,PAK3,STXBP1,RILPL1 |
| GO:1901214 | regulation of neuron death | 0.012661254 | 29 | EGLN1,FYN,NQO1,EIF4G1,BRAF,HRAS,NCOA7,CLN8,RETREG1,SYNGAP1,GRIK2,CHP1,CD200,RHOA,SNCG,VPS35,SNCB,CTNNB1,SNCA,CORO1A,MAPT,NEFL,EPHB1,GCLC,GBA1,G6PD,PAK3,STXBP1,RILPL1 |
| GO:0016239 | positive regulation of macroautophagy | 0.012705354 | 11 | SLC25A4,RALB,MAP3K7,SPTLC1,PIP4K2B,C9orf72,SH3GLB1,TSC2,SLC25A5,VDAC1,LRSAM1 |
| GO:0042135 | neurotransmitter catabolic process | 0.014887656 | 4 | MAOA,MAOB,COMT,ALDH5A1 |
| GO:0062237 | protein localization to postsynapse | 0.017693299 | 8 | NPTX1,HRAS,RAB11A,GRIN2C,CPLX1,VPS35,RAP1A,STX1B |
| GO:0021860 | pyramidal neuron development | 0.019631189 | 4 | SLC4A10,ZMIZ1,PLXNA3,DCLK2 |
| GO:1905383 | protein localization to presynapse | 0.019631189 | 4 | MAPK8IP3,MAP1A,HSPB1,KIF5A |
| GO:0050769 | positive regulation of neurogenesis | 0.019631189 | 22 | PLXNA1,ROBO2,BRAF,PLXNC1,MAP2K1,SHH,SLC7A5,TENM4,MAP6,PAFAH1B1,SYNJ1,PLXNA4,CTNNB1,SHTN1,GDI1,MAPT,NEFL,PLXNA3,CAMK2B,LRP2,RUFY3,PAK3 |
| GO:0014059 | regulation of dopamine secretion | 0.022087657 | 7 | SYT1,SYT12,SYT17,SYT13,SNCG,GABBR1,SNCA |
| GO:0031644 | regulation of nervous system process | 0.024961518 | 16 | NRXN2,NPTX1,CELF4,NPTX2,SCN11A,FMR1,FIG4,TENM4,GRIN2C,FGF12,CTSC,STX1A,HOMER1,TPPP,STX1B,GBA1 |
| GO:0030900 | forebrain development | 0.025796343 | 32 | DCLK1,DNAJB1,PLXNA1,ROBO2,FYN,SLC4A10,SHH,ALK,CEP120,RHOA,ZMIZ1,DNAH5,BMERB1,CNP,PAFAH1B1,RAC3,SLC8A1,PLXNA4,PGAP1,PCSK1,TH,CTNNB1,TUBB2A,ATAT1,NEFL,PLXNA3,AGTPBP1,DCLK2,YWHAE,LRP2,PLCB1,RTN4 |
| GO:0099054 | presynapse assembly | 0.025902563 | 8 | ARF6,BSN,EIF4G1,SDCBP,FARP1,VPS35,SNCA,EFNB3 |
| GO:0051962 | positive regulation of nervous system development | 0.037971184 | 24 | PLXNA1,ROBO2,BRAF,PLXNC1,DLG5,MAP2K1,SHH,SLC7A5,TENM4,MAP6,PAFAH1B1,SYNJ1,PLXNA4,CTNNB1,SHTN1,GDI1,MAPT,NEFL,PLXNA3,EPHB1,CAMK2B,LRP2,RUFY3,PAK3 |
| GO:0099172 | presynapse organization | 0.037988154 | 8 | ARF6,BSN,EIF4G1,SDCBP,FARP1,VPS35,SNCA,EFNB3 |
| GO:0021953 | central nervous system neuron differentiation | 0.043877629 | 17 | DCLK1,PLXNA1,CLN8,SLC4A10,SHH,HSP90AA1,SLC25A46,ZMIZ1,PAFAH1B1,RAC3,PLXNA4,SPOCK1,MAPT,PLXNA3,EPHB1,AGTPBP1,DCLK2 |
| GO:0099174 | regulation of presynapse organization | 0.049864867 | 6 | ARF6,EIF4G1,FARP1,VPS35,SNCA,EFNB3 |
| GO:1905606 | regulation of presynapse assembly | 0.049864867 | 6 | ARF6,EIF4G1,FARP1,VPS35,SNCA,EFNB3 |
| GO:0021884 | forebrain neuron development | 0.049864867 | 5 | PLXNA1,SLC4A10,ZMIZ1,PLXNA3,DCLK2 |
