# Supplementary material for: The cellular and extracellular proteomic signature of human dopaminergic neurons carrying the LRRK2 G2019S mutation
Source: Front Neurosci. 2024 Dec 12;18:1502246. doi: 10.3389/fnins.2024.1502246 (PMC11669673; doi:10.3389/fnins.2024.1502246)
Supplement: Supplementary file 10 [file Table_7.DOCX]

Supplemental Table S7. GO enrichment analysis for biological processes of the cellular proteome downregulated in L1 G2019S hDaNs.

| **GO:ID** | **description** | **adjusted**  **p-value** | **protein count** | **names** |
| --- | --- | --- | --- | --- |
| GO:0006397 | mRNA processing | 2.82E-09 | 63 | DDX41/FXR1/CDK9/USP39/U2AF2/SF3A1/APP/SMU1/TCERG1/PAXBP1/SNU13/SNRPC/RRP1B/PABPC1/PAPOLA/CWC27/DBR1/CPSF1/KDM1A/CRNKL1/DHX38/IWS1/SNRPB/NUDT21/PRPF6/PAN2/SNRPA/DDX39A/SNRPF/PNPT1/RBM4/RPRD1B/CSTF1/TXNL4A/RBM27/SRRM1/XAB2/GEMIN4/AAR2/LSM4/LSM5/TRUB1/SNRPB2/VIRMA/ALKBH5/SNRPD2/NUP98/RPUSD2/ARL6IP4/PRPF3/DAZAP1/RBM14/GPKOW/THOC2/RBM24/METTL16/RALY/SNRPD3/PNN/SART1/PCBP4/SON/PUS1 |
| GO:0008380 | RNA splicing | 6.42E-09 | 59 | DDX41/FXR1/TRRAP/TRPT1/USP39/U2AF2/SF3A1/AHNAK/SMU1/TCERG1/PAXBP1/SNU13/SNRPC/RRP1B/FUS/PABPC1/CWC27/DBR1/KDM1A/CRNKL1/DHX38/IWS1/SNRPB/PRPF6/SNRPA/DDX39A/SNRPF/RBM4/TXNL4A/SRRM1/XAB2/GEMIN4/AAR2/LSM4/LSM5/SNRPB2/VIRMA/SNRPD2/NUP98/ARL6IP4/PRPF3/RBM12B/ZNF638/DAZAP1/RBM14/GPKOW/THOC2/RBM24/METTL16/RALY/SNRPD3/PNN/SART1/PCBP4/SON/USB1/PUS1/FAM98A/SLC38A2 |
| GO:0044270 | cellular nitrogen compound catabolic process | 7.37E-08 | 58 | FXR1/EXOSC6/CNOT6/ELAVL1/EXOSC8/CNOT7/HNRNPD/SUCLG2/ITPA/IGF2BP1/CNOT3/SAMD4B/UPF2/AGO1/FEN1/CARHSP1/FUS/GTPBP1/PABPC1/YTHDF3/PAIP1/ROCK1/NUDT5/ZC3H4/TUT4/PAN2/DIS3/PYM1/PNPT1/PELO/UROD/NUDT8/SMG7/AGO2/LIN28A/LSM4/ATM/NUDT1/LSM5/PARN/TST/TDG/ALKBH5/DNASE1L1/DXO/DFFB/DCTPP1/RBM24/METTL16/PCBP4/EXOSC3/DNASE2/TENT5A/CIDEA/TET1/CNOT10/LIN28B/QPRT |
| GO:0034655 | nucleobase-containing compound catabolic process | 1.51E-07 | 54 | FXR1/EXOSC6/CNOT6/ELAVL1/EXOSC8/CNOT7/HNRNPD/SUCLG2/ITPA/IGF2BP1/CNOT3/SAMD4B/UPF2/AGO1/FEN1/CARHSP1/FUS/GTPBP1/PABPC1/YTHDF3/PAIP1/ROCK1/NUDT5/ZC3H4/TUT4/PAN2/DIS3/PYM1/PNPT1/PELO/NUDT8/SMG7/AGO2/LIN28A/LSM4/ATM/NUDT1/LSM5/PARN/TDG/ALKBH5/DNASE1L1/DXO/DFFB/DCTPP1/RBM24/METTL16/PCBP4/EXOSC3/DNASE2/TENT5A/CIDEA/CNOT10/LIN28B |
| GO:0046700 | heterocycle catabolic process | 1.51E-07 | 57 | FXR1/EXOSC6/CNOT6/ELAVL1/EXOSC8/CNOT7/HNRNPD/SUCLG2/ITPA/IGF2BP1/CNOT3/SAMD4B/UPF2/AGO1/FEN1/CARHSP1/FUS/GTPBP1/PABPC1/YTHDF3/PAIP1/ROCK1/NUDT5/ZC3H4/TUT4/PAN2/DIS3/PYM1/PNPT1/PELO/UROD/NUDT8/SMG7/AGO2/LIN28A/LSM4/ATM/NUDT1/LSM5/PARN/TDG/ALKBH5/DNASE1L1/DXO/DFFB/DCTPP1/RBM24/METTL16/PCBP4/EXOSC3/DNASE2/TENT5A/CIDEA/TET1/CNOT10/LIN28B/QPRT |
| GO:0006402 | mRNA catabolic process | 2.01E-07 | 39 | FXR1/EXOSC6/CNOT6/ELAVL1/EXOSC8/CNOT7/HNRNPD/IGF2BP1/CNOT3/SAMD4B/UPF2/AGO1/CARHSP1/FUS/GTPBP1/PABPC1/YTHDF3/PAIP1/ROCK1/TUT4/PAN2/DIS3/PYM1/PNPT1/PELO/SMG7/AGO2/LSM4/ATM/LSM5/PARN/ALKBH5/DXO/RBM24/METTL16/PCBP4/EXOSC3/TENT5A/CNOT10 |
| GO:0006401 | RNA catabolic process | 2.50E-07 | 43 | FXR1/EXOSC6/CNOT6/ELAVL1/EXOSC8/CNOT7/HNRNPD/IGF2BP1/CNOT3/SAMD4B/UPF2/AGO1/FEN1/CARHSP1/FUS/GTPBP1/PABPC1/YTHDF3/PAIP1/ROCK1/ZC3H4/TUT4/PAN2/DIS3/PYM1/PNPT1/PELO/SMG7/AGO2/LIN28A/LSM4/ATM/LSM5/PARN/ALKBH5/DXO/RBM24/METTL16/PCBP4/EXOSC3/TENT5A/CNOT10/LIN28B |
| GO:0000377 | RNA splicing, via transesterification reactions with bulged adenosine as nucleophile | 3.17E-07 | 43 | DDX41/FXR1/USP39/U2AF2/SF3A1/SMU1/PAXBP1/SNU13/SNRPC/PABPC1/CWC27/DBR1/KDM1A/CRNKL1/DHX38/SNRPB/PRPF6/SNRPA/DDX39A/SNRPF/RBM4/TXNL4A/SRRM1/XAB2/GEMIN4/AAR2/LSM4/LSM5/SNRPB2/SNRPD2/NUP98/PRPF3/DAZAP1/RBM14/GPKOW/RBM24/METTL16/RALY/SNRPD3/PNN/SART1/PCBP4/SON |
| GO:0000398 | mRNA splicing, via spliceosome | 3.17E-07 | 43 | DDX41/FXR1/USP39/U2AF2/SF3A1/SMU1/PAXBP1/SNU13/SNRPC/PABPC1/CWC27/DBR1/KDM1A/CRNKL1/DHX38/SNRPB/PRPF6/SNRPA/DDX39A/SNRPF/RBM4/TXNL4A/SRRM1/XAB2/GEMIN4/AAR2/LSM4/LSM5/SNRPB2/SNRPD2/NUP98/PRPF3/DAZAP1/RBM14/GPKOW/RBM24/METTL16/RALY/SNRPD3/PNN/SART1/PCBP4/SON |
| GO:0000956 | nuclear-transcribed mRNA catabolic process | 3.17E-07 | 25 | EXOSC6/CNOT6/EXOSC8/CNOT7/HNRNPD/IGF2BP1/CNOT3/SAMD4B/UPF2/AGO1/PABPC1/PAIP1/TUT4/PAN2/DIS3/PYM1/PELO/SMG7/AGO2/LSM4/ATM/PARN/DXO/EXOSC3/CNOT10 |
| GO:0000375 | RNA splicing, via transesterification reactions | 4.21E-07 | 43 | DDX41/FXR1/USP39/U2AF2/SF3A1/SMU1/PAXBP1/SNU13/SNRPC/PABPC1/CWC27/DBR1/KDM1A/CRNKL1/DHX38/SNRPB/PRPF6/SNRPA/DDX39A/SNRPF/RBM4/TXNL4A/SRRM1/XAB2/GEMIN4/AAR2/LSM4/LSM5/SNRPB2/SNRPD2/NUP98/PRPF3/DAZAP1/RBM14/GPKOW/RBM24/METTL16/RALY/SNRPD3/PNN/SART1/PCBP4/SON |
| GO:0034470 | ncRNA processing | 1.14E-06 | 50 | QTRT1/TRPT1/EXOSC6/DDX21/TRNT1/EXOSC8/AARS2/SNU13/RPS14/OSGEP/AGO1/PELP1/CDKAL1/MOCS3/TRMT1/BOP1/THUMPD1/RPS28/RPP30/INTS10/TUT4/WDR12/NOB1/DIS3/NSUN5/EIF6/TRMT6/INTS4/AGO2/LIN28A/GEMIN4/PARN/TRUB1/LAS1L/YRDC/TSR1/RPUSD2/GTPBP3/OSGEPL1/METTL16/SART1/RRP12/EXOSC3/POP7/NOLC1/USB1/PUS1/FAM98A/LIN28B/SEPSECS |
| GO:1903311 | regulation of mRNA metabolic process | 1.14E-06 | 42 | FXR1/CDK9/U2AF2/CNOT6/ELAVL1/EXOSC8/CNOT7/HNRNPD/SMU1/IGF2BP1/CNOT3/SAMD4B/CARHSP1/FUS/GTPBP1/PABPC1/YTHDF3/PAIP1/ROCK1/PAPOLA/IWS1/NUDT21/TUT4/PAN2/SNRPA/DIS3/PNPT1/RBM4/SRRM1/AGO2/PARN/VIRMA/ALKBH5/NUP98/DAZAP1/RBM24/METTL16/PCBP4/EXOSC3/SON/TENT5A/CNOT10 |
| GO:0050779 | RNA destabilization | 6.75E-06 | 23 | CNOT6/EXOSC8/CNOT7/HNRNPD/IGF2BP1/CNOT3/SAMD4B/PABPC1/YTHDF3/PAIP1/ROCK1/TUT4/PAN2/DIS3/PNPT1/AGO2/PARN/DXO/RBM24/METTL16/EXOSC3/CNOT10/LIN28B |
| GO:0022613 | ribonucleoprotein complex biogenesis | 8.12E-06 | 51 | USP39/EXOSC6/DDX21/SF3A1/EXOSC8/SNU13/RPS14/SNRPC/NUP88/AGO1/CUL4A/PELP1/RPL13A/BOP1/EIF3K/RPS28/RPP30/CRNKL1/GRWD1/SNRPB/NUDT21/PRPF6/WDR12/MYBBP1A/NOB1/DIS3/SNRPF/NSUN5/EIF6/CUL4B/TXNL4A/AGO2/XAB2/GEMIN4/AAR2/LSM4/ATM/SNRPB2/LAS1L/SNRPD2/TSR1/RPUSD2/PRPF3/GLUL/METTL16/SNRPD3/SART1/RRP12/EXOSC3/POP7/NOLC1 |
| GO:0043487 | regulation of RNA stability | 9.67E-06 | 30 | FXR1/CNOT6/ELAVL1/EXOSC8/CNOT7/HNRNPD/IGF2BP1/CNOT3/SAMD4B/CARHSP1/FUS/PABPC1/YTHDF3/PAIP1/ROCK1/TUT4/PAN2/DIS3/PNPT1/AGO2/PARN/ALKBH5/DXO/RBM24/METTL16/PCBP4/EXOSC3/TENT5A/CNOT10/LIN28B |
| GO:0031123 | RNA 3'-end processing | 1.38E-05 | 20 | CDK9/EXOSC6/TRNT1/EXOSC8/APP/PABPC1/PAPOLA/CPSF1/NUDT21/TUT4/SNRPA/PNPT1/RPRD1B/CSTF1/LIN28A/PARN/VIRMA/EXOSC3/USB1/LIN28B |
| GO:0000288 | nuclear-transcribed mRNA catabolic process, deadenylation-dependent decay | 1.71E-05 | 16 | CNOT6/EXOSC8/CNOT7/HNRNPD/IGF2BP1/CNOT3/SAMD4B/PABPC1/PAIP1/TUT4/PAN2/DIS3/AGO2/PARN/EXOSC3/CNOT10 |
| GO:0006399 | tRNA metabolic process | 2.19E-05 | 28 | QTRT1/TRPT1/HARS2/TRNT1/EXOSC8/AARS2/OSGEP/DTD1/CDKAL1/MOCS3/EPRS1/TRMT1/GTF3C2/THUMPD1/RPP30/YARS1/TRMT6/TRUB1/CARS2/YRDC/GTPBP3/OSGEPL1/VARS2/EXOSC3/POP7/PUS1/FAM98A/SEPSECS |
| GO:0061014 | positive regulation of mRNA catabolic process | 2.19E-05 | 22 | CNOT6/EXOSC8/CNOT7/HNRNPD/IGF2BP1/CNOT3/SAMD4B/GTPBP1/PABPC1/YTHDF3/PAIP1/ROCK1/TUT4/PAN2/DIS3/PNPT1/AGO2/PARN/RBM24/METTL16/EXOSC3/CNOT10 |
| GO:0043488 | regulation of mRNA stability | 2.57E-05 | 28 | FXR1/CNOT6/ELAVL1/EXOSC8/CNOT7/HNRNPD/IGF2BP1/CNOT3/SAMD4B/CARHSP1/FUS/PABPC1/YTHDF3/PAIP1/ROCK1/TUT4/PAN2/DIS3/PNPT1/AGO2/PARN/ALKBH5/RBM24/METTL16/PCBP4/EXOSC3/TENT5A/CNOT10 |
| GO:0048193 | Golgi vesicle transport | 2.62E-05 | 36 | SEC24B/COG4/COG3/TMED4/TRAPPC10/ERGIC3/GBF1/TMED7/TMED1/ARFGEF2/SCAMP1/SEC24D/SEC24C/STX6/COPZ1/COG6/GOLT1B/GOLGA4/COPE/CTAGE15/SEC24A/VAPB/COG7/SEC31A/COG1/GOSR1/TBC1D20/ARF4/KDELR3/CCDC91/ARFGAP3/SORT1/ERGIC2/CCDC93/SNX2/VAPA |
| GO:0006888 | endoplasmic reticulum to Golgi vesicle-mediated transport | 3.36E-05 | 22 | SEC24B/COG3/TMED4/TRAPPC10/ERGIC3/GBF1/TMED7/TMED1/SEC24D/SEC24C/GOLT1B/COPE/CTAGE15/SEC24A/VAPB/SEC31A/GOSR1/TBC1D20/ARF4/KDELR3/ERGIC2/VAPA |
| GO:0061013 | regulation of mRNA catabolic process | 3.89E-05 | 29 | FXR1/CNOT6/ELAVL1/EXOSC8/CNOT7/HNRNPD/IGF2BP1/CNOT3/SAMD4B/CARHSP1/FUS/GTPBP1/PABPC1/YTHDF3/PAIP1/ROCK1/TUT4/PAN2/DIS3/PNPT1/AGO2/PARN/ALKBH5/RBM24/METTL16/PCBP4/EXOSC3/TENT5A/CNOT10 |
| GO:1903313 | positive regulation of mRNA metabolic process | 3.98E-05 | 25 | CNOT6/EXOSC8/CNOT7/HNRNPD/IGF2BP1/CNOT3/SAMD4B/GTPBP1/PABPC1/YTHDF3/PAIP1/ROCK1/NUDT21/TUT4/PAN2/DIS3/PNPT1/AGO2/PARN/NUP98/DAZAP1/RBM24/METTL16/EXOSC3/CNOT10 |
| GO:2000113 | negative regulation of cellular macromolecule biosynthetic process | 3.98E-05 | 36 | FXR1/ACOT8/CNOT6/EXOSC8/CNOT7/HNRNPD/IGF2BP1/DBI/CNOT3/SAMD4B/AGO1/PABPC1/YTHDF3/PAIP1/ROCK1/SHMT2/RPL13A/EPRS1/PCIF1/TUT4/PAN2/DIS3/PNPT1/EIF6/RBM4/SHMT1/AGO2/LIN28A/PARN/UNK/EIF4E2/RBM24/METTL16/EXOSC3/CNOT10/EIF4EBP1 |
| GO:0061157 | mRNA destabilization | 3.98E-05 | 21 | CNOT6/EXOSC8/CNOT7/HNRNPD/IGF2BP1/CNOT3/SAMD4B/PABPC1/YTHDF3/PAIP1/ROCK1/TUT4/PAN2/DIS3/PNPT1/AGO2/PARN/RBM24/METTL16/EXOSC3/CNOT10 |
| GO:0017148 | negative regulation of translation | 3.98E-05 | 34 | FXR1/CNOT6/EXOSC8/CNOT7/HNRNPD/IGF2BP1/CNOT3/SAMD4B/AGO1/PABPC1/YTHDF3/PAIP1/ROCK1/SHMT2/RPL13A/EPRS1/PCIF1/TUT4/PAN2/DIS3/PNPT1/EIF6/RBM4/SHMT1/AGO2/LIN28A/PARN/UNK/EIF4E2/RBM24/METTL16/EXOSC3/CNOT10/EIF4EBP1 |
| GO:0070085 | glycosylation | 7.20E-05 | 31 | COG4/COG3/B3GLCT/DPY19L3/POGLUT2/MOGS/B4GALT5/GXYLT1/TMTC3/COG6/ALG10/TMEM59/MAN2A2/PSEN1/GFPT1/UBE2J1/COG7/POGLUT3/COG1/POFUT2/B4GALT7/B3GALT5/ALG12/PMM2/MGAT2/POMGNT1/ALG6/EXT1/DOLPP1/TET1/KRTCAP2 |
| GO:0009451 | RNA modification | 0.000116523 | 25 | QTRT1/AARS2/OSGEP/CDKAL1/MOCS3/TRMT1/THUMPD1/SNRPB/PCIF1/SNRPF/NSUN5/TRMT6/PARN/TRUB1/VIRMA/ALKBH5/YRDC/SNRPD2/RPUSD2/GTPBP3/OSGEPL1/METTL16/SNRPD3/PUS1/SEPSECS |
| GO:0034249 | negative regulation of amide metabolic process | 0.000122076 | 35 | FXR1/CNOT6/EXOSC8/CNOT7/HNRNPD/IGF2BP1/CNOT3/SAMD4B/AGO1/PABPC1/SPON1/YTHDF3/PAIP1/ROCK1/SHMT2/RPL13A/EPRS1/PCIF1/TUT4/PAN2/DIS3/PNPT1/EIF6/RBM4/SHMT1/AGO2/LIN28A/PARN/UNK/EIF4E2/RBM24/METTL16/EXOSC3/CNOT10/EIF4EBP1 |
| GO:0090305 | nucleic acid phosphodiester bond hydrolysis | 0.000200131 | 30 | CNOT6/EXOSC8/CNOT7/FEN1/PLD3/DBR1/BOP1/CPSF1/RAD9A/RPP30/N4BP1/NUDT21/PAN2/NOB1/DIS3/PNPT1/PELO/AGO2/PARN/POLG/LAS1L/DNASE1L1/TSR1/DXO/DFFB/ENDOU/EXOSC3/POP7/USB1/DNASE2 |
| GO:0000289 | nuclear-transcribed mRNA poly(A) tail shortening | 0.000309888 | 10 | CNOT6/CNOT7/CNOT3/SAMD4B/PABPC1/TUT4/PAN2/AGO2/PARN/CNOT10 |
| GO:0006486 | protein glycosylation | 0.000319716 | 28 | COG3/B3GLCT/DPY19L3/POGLUT2/MOGS/B4GALT5/GXYLT1/TMTC3/ALG10/TMEM59/MAN2A2/PSEN1/GFPT1/UBE2J1/COG7/POGLUT3/POFUT2/B4GALT7/B3GALT5/ALG12/PMM2/MGAT2/POMGNT1/ALG6/EXT1/DOLPP1/TET1/KRTCAP2 |
| GO:0043413 | macromolecule glycosylation | 0.000319716 | 28 | COG3/B3GLCT/DPY19L3/POGLUT2/MOGS/B4GALT5/GXYLT1/TMTC3/ALG10/TMEM59/MAN2A2/PSEN1/GFPT1/UBE2J1/COG7/POGLUT3/POFUT2/B4GALT7/B3GALT5/ALG12/PMM2/MGAT2/POMGNT1/ALG6/EXT1/DOLPP1/TET1/KRTCAP2 |
| GO:0071826 | ribonucleoprotein complex subunit organization | 0.000319716 | 29 | USP39/SF3A1/SNU13/RPS14/SNRPC/AGO1/KLC1/RPL13A/BOP1/EIF3K/RPS28/CRNKL1/SNRPB/NUDT21/PRPF6/SNRPF/EIF6/TXNL4A/AGO2/XAB2/GEMIN4/AAR2/LSM4/ATM/SNRPB2/SNRPD2/PRPF3/SNRPD3/SART1 |
| GO:0022618 | ribonucleoprotein complex assembly | 0.000471393 | 28 | USP39/SF3A1/SNU13/RPS14/SNRPC/AGO1/RPL13A/BOP1/EIF3K/RPS28/CRNKL1/SNRPB/NUDT21/PRPF6/SNRPF/EIF6/TXNL4A/AGO2/XAB2/GEMIN4/AAR2/LSM4/ATM/SNRPB2/SNRPD2/PRPF3/SNRPD3/SART1 |
| GO:2000765 | regulation of cytoplasmic translation | 0.000532137 | 9 | HNRNPD/IGF2BP1/PABPC1/CNBP/PAIP1/RPL13A/LIN28A/UNK/RBM24 |
| GO:0006913 | nucleocytoplasmic transport | 0.000541597 | 35 | UFM1/KPNA6/DDX19B/ELAVL1/NUP133/RAE1/KPNA1/UPF2/NUP88/CDAN1/TNPO1/XPO7/NUTF2/NUP214/STK4/PSEN1/IPO13/ANP32A/IWS1/NUP160/ANP32B/DDX39A/EIF6/RBM4/NUP210/SMG7/AKAP13/ALKBH5/NUP98/THOC2/RANBP3/NOLC1/IPO11/PPP1R10/FBXO22 |
| GO:0051169 | nuclear transport | 0.000541597 | 35 | UFM1/KPNA6/DDX19B/ELAVL1/NUP133/RAE1/KPNA1/UPF2/NUP88/CDAN1/TNPO1/XPO7/NUTF2/NUP214/STK4/PSEN1/IPO13/ANP32A/IWS1/NUP160/ANP32B/DDX39A/EIF6/RBM4/NUP210/SMG7/AKAP13/ALKBH5/NUP98/THOC2/RANBP3/NOLC1/IPO11/PPP1R10/FBXO22 |
| GO:0006900 | vesicle budding from membrane | 0.000581565 | 15 | SEC24B/VPS4A/TRAPPC10/GBF1/SEC24D/CHMP4B/SEC24C/SEC24A/VAPB/CHMP5/SEC31A/TBC1D20/ARFGAP3/CHMP4A/VAPA |
| GO:0009101 | glycoprotein biosynthetic process | 0.001008867 | 34 | COG3/ACOT8/UGDH/B3GLCT/DPY19L3/POGLUT2/MOGS/B4GALT5/GXYLT1/TMTC3/FAM20B/ALG10/TMEM59/BMPR2/MAN2A2/PSEN1/GFPT1/UBE2J1/COG7/POGLUT3/POFUT2/AGO2/B4GALT7/B3GALT5/ALG12/PMM2/MGAT2/POMGNT1/ALG6/EXT1/CANT1/DOLPP1/TET1/KRTCAP2 |
| GO:0034661 | ncRNA catabolic process | 0.001328287 | 11 | EXOSC6/EXOSC8/ZC3H4/TUT4/DIS3/PNPT1/PELO/LIN28A/PARN/EXOSC3/LIN28B |
| GO:0090501 | RNA phosphodiester bond hydrolysis | 0.001567062 | 21 | CNOT6/EXOSC8/CNOT7/FEN1/DBR1/BOP1/CPSF1/RPP30/N4BP1/NUDT21/PAN2/NOB1/DIS3/PNPT1/AGO2/PARN/TSR1/ENDOU/EXOSC3/POP7/USB1 |
| GO:0008033 | tRNA processing | 0.001706731 | 19 | QTRT1/TRPT1/TRNT1/AARS2/OSGEP/CDKAL1/MOCS3/TRMT1/THUMPD1/RPP30/TRMT6/TRUB1/YRDC/GTPBP3/OSGEPL1/POP7/PUS1/FAM98A/SEPSECS |
| GO:0006891 | intra-Golgi vesicle-mediated transport | 0.001865805 | 9 | COG4/COG3/TRAPPC10/COPZ1/COG6/COPE/COG7/COG1/GOSR1 |
| GO:0000387 | spliceosomal snRNP assembly | 0.002216912 | 11 | SNRPC/SNRPB/PRPF6/SNRPF/GEMIN4/AAR2/LSM4/SNRPD2/PRPF3/SNRPD3/SART1 |
| GO:0090110 | COPII-coated vesicle cargo loading | 0.003370861 | 6 | SEC24B/SEC24D/SEC24C/SEC24A/SEC31A/TBC1D20 |
| GO:2000767 | positive regulation of cytoplasmic translation | 0.003370861 | 6 | HNRNPD/IGF2BP1/PABPC1/CNBP/PAIP1/LIN28A |
| GO:0006457 | protein folding | 0.003439547 | 25 | CLPX/FKBP5/PDIA4/MOGS/FKBP2/DNAJB4/PDCD5/PDIA5/CWC27/MESD/QSOX2/HSPA14/NFYC/DNAJC3/PDIA6/PDRG1/PFDN4/CRTAP/POFUT2/ALG12/SDF2/LYRM7/ANP32E/HYPK/PDIA2 |
| GO:0006403 | RNA localization | 0.004472767 | 23 | DDX19B/NUP133/IGF2BP1/RAE1/UPF2/NUP88/NUTF2/NUP214/IWS1/PRPF6/NUP160/DDX39A/PNPT1/NUP210/SMG7/ATM/PARN/TST/ALKBH5/NUP98/CETN2/THOC2/FUBP3 |
| GO:0002181 | cytoplasmic translation | 0.004677227 | 20 | RPL28/RPL4/HNRNPD/RPS14/RPL36/IGF2BP1/PABPC1/CNBP/PAIP1/RPL21/RPL13A/EIF3K/RPS28/RBM4/LIN28A/EIF4A1/DPH1/UNK/RBM24/RPL37A |
| GO:0031054 | pre-miRNA processing | 0.004677227 | 6 | AGO1/TUT4/AGO2/LIN28A/TRUB1/LIN28B |
| GO:0071025 | RNA surveillance | 0.004677227 | 6 | EXOSC6/EXOSC8/ZC3H4/PELO/DXO/EXOSC3 |
| GO:0031124 | mRNA 3'-end processing | 0.004677227 | 11 | CDK9/APP/PABPC1/PAPOLA/CPSF1/NUDT21/SNRPA/PNPT1/RPRD1B/CSTF1/VIRMA |
| GO:0006406 | mRNA export from nucleus | 0.005346401 | 12 | DDX19B/NUP133/RAE1/UPF2/NUP88/NUP214/IWS1/NUP160/DDX39A/SMG7/ALKBH5/THOC2 |
| GO:0042254 | ribosome biogenesis | 0.005346401 | 30 | EXOSC6/DDX21/EXOSC8/SNU13/RPS14/NUP88/CUL4A/PELP1/BOP1/RPS28/RPP30/GRWD1/WDR12/MYBBP1A/NOB1/DIS3/NSUN5/EIF6/CUL4B/GEMIN4/LAS1L/TSR1/RPUSD2/GLUL/METTL16/SART1/RRP12/EXOSC3/POP7/NOLC1 |
| GO:0009259 | ribonucleotide metabolic process | 0.005592847 | 41 | LDHA/DMAC2L/NDUFA8/ACOT8/CLPX/PGK1/CAD/PAICS/APP/SUCLG2/DGUOK/ITPA/NDUFA12/GTPBP1/ATP6V0C/GPD1/NDUFB11/NDUFV2/AK2/SDHC/NUDT5/NDUFS4/PSEN1/GMPR2/IMPDH2/ADSS1/PPAT/EIF6/NUDT8/PRPS2/NME3/HK2/DGAT1/PAPSS2/MPP1/ACSF3/ACSF2/ACSL6/UCKL1/ACSS1/PANK2 |
| GO:0016072 | rRNA metabolic process | 0.005708957 | 27 | EXOSC6/DDX21/EXOSC8/SNU13/RPS14/PELP1/BOP1/GTF3C2/RPS28/RPP30/WDR12/NOB1/DIS3/NSUN5/EIF6/PELO/GEMIN4/LAS1L/TSR1/RPUSD2/MACROH2A2/METTL16/SART1/RRP12/EXOSC3/POP7/NOLC1 |
| GO:0051236 | establishment of RNA localization | 0.005708957 | 20 | DDX19B/NUP133/IGF2BP1/RAE1/UPF2/NUP88/NUTF2/NUP214/IWS1/NUP160/DDX39A/PNPT1/NUP210/SMG7/ATM/TST/ALKBH5/NUP98/CETN2/THOC2 |
| GO:0031331 | positive regulation of cellular catabolic process | 0.005708957 | 40 | CNOT6/EXOSC8/CNOT7/APP/HNRNPD/IGF2BP1/CNOT3/SNX18/SAMD4B/GTPBP1/PABPC1/GPD1/YTHDF3/PAIP1/ROCK1/ATG101/TMEM59/PSEN1/DVL1/PTK2/TUT4/PAN2/DIS3/PIK3C2A/PNPT1/SPTLC2/RAD23A/AGO2/ATM/PARN/PHKG2/BECN1/DXO/RBM24/METTL16/EXOSC3/UBQLN1/CNOT10/LIN28B/FBXO22 |
| GO:0070972 | protein localization to endoplasmic reticulum | 0.005925054 | 13 | GBF1/CHMP4B/UBL4A/SRP54/SRPRB/SRPRA/SEC61G/SEC63/SGTB/KDELR3/CHMP4A/PDIA2/VAPA |
| GO:0006620 | post-translational protein targeting to endoplasmic reticulum membrane | 0.005982305 | 6 | CHMP4B/UBL4A/SEC61G/SEC63/SGTB/CHMP4A |
| GO:0006364 | rRNA processing | 0.006327624 | 24 | EXOSC6/DDX21/EXOSC8/SNU13/RPS14/PELP1/BOP1/RPS28/RPP30/WDR12/NOB1/DIS3/NSUN5/EIF6/GEMIN4/LAS1L/TSR1/RPUSD2/METTL16/SART1/RRP12/EXOSC3/POP7/NOLC1 |
| GO:0019693 | ribose phosphate metabolic process | 0.007613166 | 41 | LDHA/DMAC2L/NDUFA8/ACOT8/CLPX/PGK1/CAD/PAICS/APP/SUCLG2/DGUOK/ITPA/NDUFA12/GTPBP1/ATP6V0C/GPD1/NDUFB11/NDUFV2/AK2/SDHC/NUDT5/NDUFS4/PSEN1/GMPR2/IMPDH2/ADSS1/PPAT/EIF6/NUDT8/PRPS2/NME3/HK2/DGAT1/PAPSS2/MPP1/ACSF3/ACSF2/ACSL6/UCKL1/ACSS1/PANK2 |
| GO:0009124 | nucleoside monophosphate biosynthetic process | 0.00790594 | 9 | CAD/PAICS/DGUOK/IMPDH2/ADSS1/PPAT/SHMT1/PRPS2/UCKL1 |
| GO:0051028 | mRNA transport | 0.007986623 | 17 | DDX19B/NUP133/IGF2BP1/RAE1/UPF2/NUP88/NUTF2/NUP214/IWS1/NUP160/DDX39A/NUP210/SMG7/ALKBH5/NUP98/CETN2/THOC2 |
| GO:0046112 | nucleobase biosynthetic process | 0.007986623 | 6 | CAD/PAICS/SHMT2/PPAT/SHMT1/CPS1 |
| GO:0009150 | purine ribonucleotide metabolic process | 0.008227459 | 39 | LDHA/DMAC2L/NDUFA8/ACOT8/CLPX/PGK1/PAICS/APP/SUCLG2/DGUOK/ITPA/NDUFA12/GTPBP1/ATP6V0C/GPD1/NDUFB11/NDUFV2/AK2/SDHC/NUDT5/NDUFS4/PSEN1/GMPR2/IMPDH2/ADSS1/PPAT/EIF6/NUDT8/PRPS2/NME3/HK2/DGAT1/PAPSS2/MPP1/ACSF3/ACSF2/ACSL6/ACSS1/PANK2 |
| GO:0006890 | retrograde vesicle-mediated transport, Golgi to endoplasmic reticulum | 0.008227459 | 10 | COG4/COG3/ERGIC3/GBF1/COPZ1/COPE/COG7/ARF4/KDELR3/ERGIC2 |
| GO:0006400 | tRNA modification | 0.008302003 | 14 | QTRT1/AARS2/OSGEP/CDKAL1/MOCS3/TRMT1/THUMPD1/TRMT6/TRUB1/YRDC/GTPBP3/OSGEPL1/PUS1/SEPSECS |
| GO:0009650 | UV protection | 0.008551799 | 5 | GPX1/FEN1/ERCC1/ERCC3/ERCC4 |
| GO:0009112 | nucleobase metabolic process | 0.008552564 | 8 | CAD/PAICS/SHMT2/KDM1A/GMPR2/PPAT/SHMT1/CPS1 |
| GO:0090503 | RNA phosphodiester bond hydrolysis, exonucleolytic | 0.008552564 | 9 | CNOT6/EXOSC8/CNOT7/PAN2/DIS3/PNPT1/PARN/EXOSC3/USB1 |
| GO:0009100 | glycoprotein metabolic process | 0.008607035 | 36 | COG3/ACOT8/UGDH/B3GLCT/DPY19L3/POGLUT2/MOGS/B4GALT5/GUSB/ADAMTS12/GXYLT1/TMTC3/FAM20B/ALG10/TMEM59/BMPR2/MAN2A2/PSEN1/GFPT1/UBE2J1/COG7/POGLUT3/POFUT2/AGO2/B4GALT7/B3GALT5/ALG12/PMM2/MGAT2/POMGNT1/ALG6/EXT1/CANT1/DOLPP1/TET1/KRTCAP2 |
| GO:0006289 | nucleotide-excision repair | 0.009143238 | 13 | DPF2/SMARCC2/CUL4A/ACTL6A/PBRM1/RPA2/RAD23A/ERCC1/XAB2/SMARCC1/ERCC3/CETN2/ERCC4 |
| GO:0009141 | nucleoside triphosphate metabolic process | 0.009475702 | 27 | LDHA/DMAC2L/NDUFA8/CLPX/PGK1/CAD/APP/DGUOK/ITPA/NDUFA12/GTPBP1/ATP6V0C/GPD1/NDUFB11/NDUFV2/AK2/SDHC/NUDT5/NDUFS4/PSEN1/IMPDH2/EIF6/NME3/HK2/DCTPP1/RRM2B/UCKL1 |
| GO:0000291 | nuclear-transcribed mRNA catabolic process, exonucleolytic | 0.009538348 | 6 | EXOSC6/CNOT6/EXOSC8/CNOT7/DIS3/EXOSC3 |
| GO:0009226 | nucleotide-sugar biosynthetic process | 0.009538348 | 6 | GNPNAT1/UGDH/MPI/GFPT1/UAP1/PMM2 |
| GO:0009126 | purine nucleoside monophosphate metabolic process | 0.009538348 | 9 | PAICS/DGUOK/AK2/GMPR2/IMPDH2/ADSS1/PPAT/PRPS2/MPP1 |
| GO:0048041 | focal adhesion assembly | 0.009682358 | 13 | CAMSAP3/RCC2/VCL/ROCK1/TAOK2/LIMS1/TLN1/PTK2/PIP5K1A/NRP1/SORBS1/BCAS3/LIMCH1 |
| GO:0050657 | nucleic acid transport | 0.009707727 | 19 | DDX19B/NUP133/IGF2BP1/RAE1/UPF2/NUP88/NUTF2/NUP214/IWS1/NUP160/DDX39A/PNPT1/NUP210/SMG7/TST/ALKBH5/NUP98/CETN2/THOC2 |
| GO:0050658 | RNA transport | 0.009707727 | 19 | DDX19B/NUP133/IGF2BP1/RAE1/UPF2/NUP88/NUTF2/NUP214/IWS1/NUP160/DDX39A/PNPT1/NUP210/SMG7/TST/ALKBH5/NUP98/CETN2/THOC2 |
| GO:0009123 | nucleoside monophosphate metabolic process | 0.010829052 | 12 | CAD/PAICS/DGUOK/AK2/GMPR2/IMPDH2/ADSS1/PPAT/SHMT1/PRPS2/MPP1/UCKL1 |
| GO:0006563 | L-serine metabolic process | 0.011188359 | 5 | PSAT1/PHGDH/PSPH/SHMT2/SHMT1 |
| GO:0043928 | exonucleolytic catabolism of deadenylated mRNA | 0.011188359 | 5 | CNOT6/EXOSC8/CNOT7/DIS3/EXOSC3 |
| GO:0006144 | purine nucleobase metabolic process | 0.012029752 | 6 | PAICS/SHMT2/KDM1A/GMPR2/PPAT/SHMT1 |
| GO:0006378 | mRNA polyadenylation | 0.013342935 | 8 | APP/PABPC1/PAPOLA/CPSF1/NUDT21/SNRPA/PNPT1/VIRMA |
| GO:0050684 | regulation of mRNA processing | 0.013694603 | 17 | FXR1/CDK9/U2AF2/SMU1/PAPOLA/IWS1/NUDT21/SNRPA/RBM4/SRRM1/VIRMA/NUP98/DAZAP1/RBM24/METTL16/PCBP4/SON |
| GO:0016050 | vesicle organization | 0.013694603 | 33 | SEC24B/OSBP/CLCN3/VPS4A/TRAPPC10/GBF1/ARFGEF2/SEC24D/ATP6V0C/CHMP4B/SEC24C/STX6/SEC24A/VAPB/HGS/CREB1/CHMP5/SHROOM2/SEC31A/GOSR1/TBC1D20/MYO7A/FHIP1B/TMEM127/ARFGAP3/RAB11A/CHMP4A/VAMP4/SORT1/TMF1/KCNE1/SYT1/VAPA |
| GO:0006405 | RNA export from nucleus | 0.013709663 | 13 | DDX19B/NUP133/RAE1/UPF2/NUP88/NUP214/IWS1/NUP160/DDX39A/SMG7/ALKBH5/NUP98/THOC2 |
| GO:0051168 | nuclear export | 0.014145388 | 19 | DDX19B/NUP133/RAE1/UPF2/NUP88/XPO7/NUTF2/NUP214/IWS1/NUP160/ANP32B/DDX39A/EIF6/SMG7/AKAP13/ALKBH5/NUP98/THOC2/RANBP3 |
| GO:0150115 | cell-substrate junction organization | 0.014994123 | 14 | CAMSAP3/RCC2/VCL/ROCK1/TAOK2/LIMS1/IQSEC1/TLN1/PTK2/PIP5K1A/NRP1/SORBS1/BCAS3/LIMCH1 |
| GO:0009199 | ribonucleoside triphosphate metabolic process | 0.014994123 | 25 | LDHA/DMAC2L/NDUFA8/CLPX/PGK1/CAD/APP/DGUOK/ITPA/NDUFA12/GTPBP1/ATP6V0C/GPD1/NDUFB11/NDUFV2/AK2/SDHC/NUDT5/NDUFS4/PSEN1/IMPDH2/EIF6/NME3/HK2/UCKL1 |
| GO:0010587 | miRNA catabolic process | 0.014994123 | 5 | TUT4/PNPT1/LIN28A/PARN/LIN28B |
| GO:0071027 | nuclear RNA surveillance | 0.014994123 | 5 | EXOSC6/EXOSC8/ZC3H4/DXO/EXOSC3 |
| GO:0045047 | protein targeting to ER | 0.015592452 | 9 | CHMP4B/UBL4A/SRP54/SRPRB/SRPRA/SEC61G/SEC63/SGTB/CHMP4A |
| GO:0150116 | regulation of cell-substrate junction organization | 0.015592452 | 11 | CAMSAP3/RCC2/VCL/ROCK1/LIMS1/IQSEC1/TLN1/PTK2/NRP1/BCAS3/LIMCH1 |
| GO:0009161 | ribonucleoside monophosphate metabolic process | 0.016865887 | 10 | CAD/PAICS/AK2/GMPR2/IMPDH2/ADSS1/PPAT/PRPS2/MPP1/UCKL1 |
| GO:0010257 | NADH dehydrogenase complex assembly | 0.016865887 | 10 | NDUFA8/NDUFAF7/OXA1L/NDUFA12/NDUFB11/NDUFS4/NDUFAF2/TMEM126A/ECSIT/FOXRED1 |
| GO:0032981 | mitochondrial respiratory chain complex I assembly | 0.016865887 | 10 | NDUFA8/NDUFAF7/OXA1L/NDUFA12/NDUFB11/NDUFS4/NDUFAF2/TMEM126A/ECSIT/FOXRED1 |
| GO:0043631 | RNA polyadenylation | 0.016891054 | 8 | APP/PABPC1/PAPOLA/CPSF1/NUDT21/SNRPA/PNPT1/VIRMA |
| GO:0009127 | purine nucleoside monophosphate biosynthetic process | 0.018056192 | 6 | PAICS/DGUOK/IMPDH2/ADSS1/PPAT/PRPS2 |
| GO:0016556 | mRNA modification | 0.020775904 | 7 | PCIF1/TRMT6/TRUB1/VIRMA/RPUSD2/METTL16/PUS1 |
| GO:0006487 | protein N-linked glycosylation | 0.021241119 | 11 | MOGS/ALG10/GFPT1/UBE2J1/B4GALT7/ALG12/PMM2/MGAT2/ALG6/DOLPP1/KRTCAP2 |
| GO:0007044 | cell-substrate junction assembly | 0.022363048 | 13 | CAMSAP3/RCC2/VCL/ROCK1/TAOK2/LIMS1/TLN1/PTK2/PIP5K1A/NRP1/SORBS1/BCAS3/LIMCH1 |
| GO:0009260 | ribonucleotide biosynthetic process | 0.022819981 | 23 | DMAC2L/NDUFA8/CAD/PAICS/DGUOK/NDUFA12/ATP6V0C/NDUFB11/NDUFV2/AK2/SDHC/NDUFS4/IMPDH2/ADSS1/PPAT/PRPS2/NME3/PAPSS2/ACSF3/ACSL6/UCKL1/ACSS1/PANK2 |
| GO:2000756 | regulation of peptidyl-lysine acetylation | 0.023796487 | 10 | DDX21/HINT2/SIN3A/TAF7/NFYC/IWS1/NFYA/MYBBP1A/SMARCA5/SMAD4 |
| GO:0015980 | energy derivation by oxidation of organic compounds | 0.023839641 | 30 | GAA/NDUFA8/OXA1L/SUCLG2/DGUOK/NDUFA12/NIPSNAP2/PHKA2/GPD1/ETFRF1/NDUFB11/NDUFV2/SDHC/SHMT2/NDUFS4/ACO1/GFPT1/MYBBP1A/PNPT1/PYGB/SORBS1/PHKG2/LYRM7/NR4A3/PPP1CB/UQCRH/UQCR11/CHCHD2/PYGM/PANK2 |
| GO:0007032 | endosome organization | 0.023843939 | 13 | CLCN3/VPS4A/ARFGEF2/ATP6V0C/CHMP4B/STX6/HGS/CHMP5/FHIP1B/TMEM127/RAB11A/CHMP4A/VAMP4 |
| GO:0016197 | endosomal transport | 0.025329671 | 24 | VPS4A/TRAPPC10/VPS26A/GBF1/SNX18/CHMP4B/STX6/GOLT1B/HGS/CHMP5/CLTCL1/GOSR1/EHD4/EIPR1/DPY30/RAB11A/CHMP4A/VAMP4/SORT1/ACAP2/CCDC93/TMEM50A/SNX2/RIC1 |
| GO:0072599 | establishment of protein localization to endoplasmic reticulum | 0.025329671 | 9 | CHMP4B/UBL4A/SRP54/SRPRB/SRPRA/SEC61G/SEC63/SGTB/CHMP4A |
| GO:0009167 | purine ribonucleoside monophosphate metabolic process | 0.025849436 | 8 | PAICS/AK2/GMPR2/IMPDH2/ADSS1/PPAT/PRPS2/MPP1 |
| GO:1903241 | U2-type prespliceosome assembly | 0.027170588 | 6 | SF3A1/SNRPB/SNRPF/SNRPB2/SNRPD2/SNRPD3 |
| GO:0015931 | nucleobase-containing compound transport | 0.027743893 | 22 | DDX19B/SLC35B1/NUP133/IGF2BP1/RAE1/UPF2/NUP88/NUTF2/NUP214/IWS1/NUP160/DDX39A/PNPT1/NUP210/ABCC4/SMG7/SLC35B2/TST/ALKBH5/NUP98/CETN2/THOC2 |
| GO:0035459 | vesicle cargo loading | 0.027743893 | 7 | SEC24B/SEC24D/SEC24C/CTAGE15/SEC24A/SEC31A/TBC1D20 |
| GO:0035065 | regulation of histone acetylation | 0.027743893 | 9 | DDX21/SIN3A/TAF7/NFYC/IWS1/NFYA/MYBBP1A/SMARCA5/SMAD4 |
| GO:0072594 | establishment of protein localization to organelle | 0.027743893 | 37 | UFM1/VPS4A/KPNA6/ELAVL1/OXA1L/NUP133/KPNA1/NUP88/TNPO1/CHMP4B/UBL4A/SRP54/NUTF2/NUP214/SRPRB/PDCD5/STK4/HGS/PSEN1/IPO13/SRPRA/SEC61G/SEC63/NMT1/SGTB/NUP98/BECN1/HK2/PXK/RAB11A/CHMP4A/MACROH2A2/SORT1/TIMM13/NOLC1/IPO11/PPP1R10 |
| GO:0051893 | regulation of focal adhesion assembly | 0.027743893 | 10 | CAMSAP3/RCC2/VCL/ROCK1/LIMS1/TLN1/PTK2/NRP1/BCAS3/LIMCH1 |
| GO:0090109 | regulation of cell-substrate junction assembly | 0.027743893 | 10 | CAMSAP3/RCC2/VCL/ROCK1/LIMS1/TLN1/PTK2/NRP1/BCAS3/LIMCH1 |
| GO:0090114 | COPII-coated vesicle budding | 0.028462642 | 8 | SEC24B/SEC24D/SEC24C/SEC24A/VAPB/SEC31A/TBC1D20/VAPA |
| GO:0009113 | purine nucleobase biosynthetic process | 0.030035947 | 4 | PAICS/SHMT2/PPAT/SHMT1 |
| GO:0046390 | ribose phosphate biosynthetic process | 0.030035947 | 23 | DMAC2L/NDUFA8/CAD/PAICS/DGUOK/NDUFA12/ATP6V0C/NDUFB11/NDUFV2/AK2/SDHC/NDUFS4/IMPDH2/ADSS1/PPAT/PRPS2/NME3/PAPSS2/ACSF3/ACSL6/UCKL1/ACSS1/PANK2 |
| GO:0009156 | ribonucleoside monophosphate biosynthetic process | 0.031559468 | 7 | CAD/PAICS/IMPDH2/ADSS1/PPAT/PRPS2/UCKL1 |
| GO:1901983 | regulation of protein acetylation | 0.031935739 | 11 | DDX21/HINT2/SIN3A/HDAC6/TAF7/NFYC/IWS1/NFYA/MYBBP1A/SMARCA5/SMAD4 |
| GO:0045727 | positive regulation of translation | 0.033441204 | 16 | FXR1/ELAVL1/HNRNPD/IGF2BP1/PABPC1/CNBP/YTHDF3/PAIP1/PCIF1/DNAJC3/PYM1/NSUN5/EIF6/RBM4/LIN28A/RCC1L |
| GO:0006541 | glutamine metabolic process | 0.038222031 | 6 | CAD/PHGDH/GFPT1/PPAT/GLUL/CPS1 |
| GO:1900151 | regulation of nuclear-transcribed mRNA catabolic process, deadenylation-dependent decay | 0.038222031 | 6 | CNOT7/HNRNPD/IGF2BP1/PABPC1/PAIP1/AGO2 |
| GO:0007030 | Golgi organization | 0.038895388 | 17 | COG4/CAMSAP3/COG3/TMED4/DYM/GBF1/TMED7/TMED1/COG6/MAP2K2/PDE4DIP/CUL7/COG7/COG1/TBC1D20/VAMP4/BCAS3 |
| GO:0009205 | purine ribonucleoside triphosphate metabolic process | 0.039834479 | 23 | LDHA/DMAC2L/NDUFA8/CLPX/PGK1/APP/DGUOK/ITPA/NDUFA12/GTPBP1/ATP6V0C/GPD1/NDUFB11/NDUFV2/AK2/SDHC/NUDT5/NDUFS4/PSEN1/IMPDH2/EIF6/NME3/HK2 |
| GO:0070934 | CRD-mediated mRNA stabilization | 0.042662147 | 4 | HNRNPD/IGF2BP1/PABPC1/PAIP1 |
| GO:0009152 | purine ribonucleotide biosynthetic process | 0.045576647 | 21 | DMAC2L/NDUFA8/PAICS/DGUOK/NDUFA12/ATP6V0C/NDUFB11/NDUFV2/AK2/SDHC/NDUFS4/IMPDH2/ADSS1/PPAT/PRPS2/NME3/PAPSS2/ACSF3/ACSL6/ACSS1/PANK2 |
| GO:0006260 | DNA replication | 0.047345682 | 25 | CDK9/CAMSAP3/MAP2K4/RBBP4/RECQL/FEN1/SIN3A/DTD1/RFC1/ACTL6A/WAPL/PCNA/GRWD1/ANKRD17/RPA2/MCMBP/BCAR3/LPIN1/NASP/RFC5/WRNIP1/POLG/RBMS1/SMARCA5/RRM2B |
| GO:0045324 | late endosome to vacuole transport | 0.049494844 | 7 | VPS4A/CHMP4B/HGS/CHMP5/BECN1/CHMP4A/TMEM50A |
| GO:1901607 | alpha-amino acid biosynthetic process | 0.049677679 | 10 | CAD/PSAT1/PHGDH/PSPH/SHMT2/BCAT1/SHMT1/APIP/GLUL/CPS1 |
| GO:0009144 | purine nucleoside triphosphate metabolic process | 0.049677679 | 23 | LDHA/DMAC2L/NDUFA8/CLPX/PGK1/APP/DGUOK/ITPA/NDUFA12/GTPBP1/ATP6V0C/GPD1/NDUFB11/NDUFV2/AK2/SDHC/NUDT5/NDUFS4/PSEN1/IMPDH2/EIF6/NME3/HK2 |
| GO:0009165 | nucleotide biosynthetic process | 0.049677679 | 26 | DMAC2L/NDUFA8/CAD/PAICS/DGUOK/NDUFA12/ATP6V0C/NDUFB11/NDUFV2/AK2/SDHC/NDUFS4/IMPDH2/ADSS1/PPAT/SHMT1/PRPS2/NME3/PAPSS2/ACSF3/ACSL6/RRM2B/UCKL1/ACSS1/PANK2/QPRT |
