# Supplementary material for: The cellular and extracellular proteomic signature of human dopaminergic neurons carrying the LRRK2 G2019S mutation
Source: Front Neurosci. 2024 Dec 12;18:1502246. doi: 10.3389/fnins.2024.1502246 (PMC11669673; doi:10.3389/fnins.2024.1502246)
Supplement: Supplementary file 11 [file Table_8.DOCX]

Supplemental Table S8. GO enrichment analysis for CNS related biological processes of the dysregulated EV and cellular proteome in L1 G2019S.

| **GO:ID** | **description** | **adjusted p-value** | **protein count** | **names** |
| --- | --- | --- | --- | --- |
| GO:0050808 | synapse organization | 0.000111966 | 15 | ACTB,AFG3L2,ARF4,DIP2A,EPHB1,ITGB1,LRFN3,NEFL,NPTX1,NRXN2,PCDHB2,RAP2A,RHOA,SDCBP,TUBB |
| GO:0021535 | cell migration in hindbrain | 0.01447385 | 3 | ATP1B2,EPHB1,ITGB1 |
| GO:0050807 | regulation of synapse organization | 0.01447385 | 8 | ARF4,EPHB1,ITGB1,LRFN3,NEFL,RAP2A,RHOA,TUBB |
| GO:0050803 | regulation of synapse structure or activity | 0.015947635 | 8 | ARF4,EPHB1,ITGB1,LRFN3,NEFL,RAP2A,RHOA,TUBB |
| GO:0099173 | postsynapse organization | 0.016668145 | 7 | ACTB,ARF4,DIP2A,EPHB1,NEFL,NPTX1,NRXN2 |
| GO:0099072 | regulation of postsynaptic membrane neurotransmitter receptor levels | 0.020531901 | 5 | AP2A1,CALY,HSP90AA1,NPTX1,RAP2A |
| GO:0007416 | synapse assembly | 0.021098181 | 7 | EPHB1,LRFN3,NPTX1,NRXN2,PCDHB2,RAP2A,SDCBP |
| GO:0098962 | regulation of postsynaptic neurotransmitter receptor activity | 0.021515475 | 3 | NPTX1,NPTX2,NRXN2 |
