# Supplementary material for: The cellular and extracellular proteomic signature of human dopaminergic neurons carrying the LRRK2 G2019S mutation
Source: Front Neurosci. 2024 Dec 12;18:1502246. doi: 10.3389/fnins.2024.1502246 (PMC11669673; doi:10.3389/fnins.2024.1502246)
Supplement: Supplementary file 12 [file Table_9.DOCX]

Supplemental Table S9. List of the 123 proteins dysregulated in both the EV and cellular proteome and the number of publications mentioning the respective protein in the context of either “Parkinson’s Disease” or “LRRK2”

| Protein | Parkinson's Disease | LRRK2 |
| --- | --- | --- |
| ACO1 | 5 | 0 |
| ACTB | 14 | 0 |
| ACVR1B | 2 | 2 |
| ADAM22 | 1 | 0 |
| AFG3L2 | 6 | 0 |
| AGO1 | 2 | 1 |
| AKR1C2 | 1 | 0 |
| ALDH5A1 | 0 | 0 |
| ANXA1 | 7 | 1 |
| AP2A1 | 0 | 0 |
| ARF4 | 0 | 0 |
| ARRB1 | 1 | 0 |
| ATP1B2 | 2 | 0 |
| ATP5F1B | 4 | 0 |
| ATP6V0C | 2 | 0 |
| CALM1 | 3 | 0 |
| CALY | 0 | 0 |
| CAMK2A | 4 | 0 |
| CANT1 | 0 | 0 |
| CBX1 | 0 | 1 |
| CBX3 | 0 | 0 |
| CD44 | 29 | 1 |
| CDSN | 1 | 3 |
| CHMP5 | 1 | 0 |
| CLEC18B | 0 | 0 |
| CLIP2 | 0 | 0 |
| COQ5 | 0 | 0 |
| CPS1 | 0 | 1 |
| CUL4A | 2 | 0 |
| DCX | 39 | 1 |
| DDAH1 | 0 | 0 |
| DIP2A | 0 | 0 |
| DNM1 | 2 | 0 |
| DSTN | 2 | 0 |
| EDIL3 | 0 | 0 |
| EEF1B2 | 0 | 1 |
| EIF4A2 | 1 | 0 |
| EPHA2 | 2 | 0 |
| EPHB1 | 4 | 0 |
| EPPK1 | 1 | 0 |
| ERH | 2 | 0 |
| EXT1 | 0 | 1 |
| FKBP15 | 0 | 0 |
| FLNC | 0 | 0 |
| GALC | 22 | 0 |
| GCAT | 2 | 0 |
| GLUL | 1 | 0 |
| GNB1 | 1 | 0 |
| GRIA1 | 5 | 2 |
| HIBADH | 0 | 0 |
| HMGB3 | 0 | 0 |
| HNRNPD | 1 | 0 |
| HNRNPR | 0 | 0 |
| HPCAL1 | 0 | 0 |
| HSP90AA1 | 3 | 1 |
| HTRA1 | 12 | 0 |
| IGFBP7 | 0 | 4 |
| ITCH | 25 | 0 |
| ITGB1 | 1 | 0 |
| KATNAL2 | 0 | 0 |
| KRT1 | 1 | 0 |
| KRT10 | 0 | 0 |
| KRT14 | 0 | 4 |
| KRT16 | 1 | 0 |
| KRT2 | 1 | 0 |
| KRT6A | 0 | 2 |
| LINGO1 | 30 | 0 |
| LRFN3 | 0 | 0 |
| MAPRE3 | 2 | 0 |
| MFGE8 | 6 | 0 |
| MUC5AC | 0 | 0 |
| MYH14 | 1 | 0 |
| NCDN | 0 | 0 |
| NECAB2 | 2 | 0 |
| NEFL | 4 | 0 |
| NPTX1 | 3 | 0 |
| NPTX2 | 9 | 2 |
| NRXN2 | 5 | 0 |
| OSBP | 0 | 0 |
| P2RX3 | 1 | 2 |
| PABPC1 | 0 | 0 |
| PAK1 | 8 | 0 |
| PCDHA4 | 2 | 2 |
| PCDHB2 | 0 | 0 |
| PDCD6 | 0 | 0 |
| PDIA6 | 1 | 0 |
| PKM | 10 | 0 |
| PLCG1 | 3 | 0 |
| POTEF | 1 | 0 |
| PPA2 | 0 | 23 |
| PPP2R5C | 1 | 0 |
| PRDX2 | 5 | 0 |
| PSMC5 | 2 | 0 |
| PTPRG | 0 | 0 |
| PYGB | 0 | 0 |
| RAP2A | 0 | 0 |
| RBBP4 | 1 | 0 |
| REEP6 | 0 | 0 |
| RHOA | 39 | 0 |
| RNMT | 0 | 0 |
| SDCBP | 0 | 1 |
| SHH | 119 | 0 |
| SLC2A1 | 12 | 0 |
| SMU1 | 0 | 0 |
| SNRPB | 0 | 2 |
| SORCS3 | 2 | 0 |
| SPON1 | 1 | 0 |
| SPRY4 | 1 | 0 |
| STMN1 | 2 | 0 |
| STX7 | 0 | 0 |
| SYNJ1 | 64 | 0 |
| SYT1 | 13 | 0 |
| TENM3 | 0 | 0 |
| TIMP1 | 16 | 0 |
| TLN1 | 2 | 0 |
| TSPAN14 | 2 | 0 |
| TUBB | 1 | 0 |
| TUBB2A | 2 | 0 |
| TUBB3 | 17 | 0 |
| U2AF2 | 1 | 1 |
| UBE2V1 | 0 | 0 |
| UGGT1 | 0 | 0 |
| VPS4A | 1 | 0 |
